# Genome-wide DNA methylation patterns reveal clinically relevant predictive and prognostic subtypes in human osteosarcoma

**DOI:** 10.1038/s42003-022-03117-1

**Published:** 2022-03-08

**Authors:** Christopher E. Lietz, Erik T. Newman, Andrew D. Kelly, David H. Xiang, Ziying Zhang, Caroline A. Luscko, Santiago A. Lozano-Calderon, David H. Ebb, Kevin A. Raskin, Gregory M. Cote, Edwin Choy, G. Petur Nielsen, Benjamin Haibe-Kains, Martin J. Aryee, Dimitrios Spentzos

**Affiliations:** 1 Department of Orthopaedic Surgery, Massachusetts General Hospital Cancer Center, Harvard Medical School, Boston, MA USA; 2 Foundation Medicine, Cambridge, MA USA; 3 Division of Pediatric Hematology/Oncology, Massachusetts General Hospital, Harvard Medical School, Boston, MA USA; 4 Division of Hematology/Oncology, Massachusetts General Hospital Cancer Center, Harvard Medical School, Boston, MA USA; 5 Department of Pathology, Massachusetts General Hospital, Harvard Medical School, Boston, MA USA; 6 Department of Medical Biophysics, Princess Margaret Cancer Centre, University of Toronto, Toronto, M5G 2M9 ON Canada

**Keywords:** Bone cancer, Biomarkers, Bone cancer, Tumour biomarkers, Epigenomics

## Abstract

Aberrant methylation of genomic DNA has been reported in many cancers. Specific DNA methylation patterns have been shown to provide clinically useful prognostic information and define molecular disease subtypes with different response to therapy and long-term outcome. Osteosarcoma is an aggressive malignancy for which approximately half of tumors recur following standard combined surgical resection and chemotherapy. No accepted prognostic factor save tumor necrosis in response to adjuvant therapy currently exists, and traditional genomic studies have thus far failed to identify meaningful clinical associations. We studied the genome-wide methylation state of primary tumors and tested how they predict patient outcomes. We discovered relative genomic hypomethylation to be strongly predictive of response to standard chemotherapy. Recurrence and survival were also associated with genomic methylation, but through more site-specific patterns. Furthermore, the methylation patterns were reproducible in three small independent clinical datasets. Downstream transcriptional, in vitro, and pharmacogenomic analysis provides insight into the clinical translation of the methylation patterns. Our findings suggest the assessment of genomic methylation may represent a strategy for stratifying patients for the application of alternative therapies.

## Abstract (web summary)

Christopher Lietz et al. examined genome-wide methylation in patient cohorts of primary osteosarcoma. Their results suggest that genomic hypomethylation is predictive of response to standard chemotherapy, and provide further insight into clinical implications of methylation patterns.

## Introduction

Osteosarcoma (OSA) is a rare primary bone tumor with high metastatic potential to spread to the lungs even after treatment. The systemic management of pediatric OSA, which includes multi-agent chemotherapy, resulted in improvements in cure rates following the adoption in the 1980s^1,2^, but has remained largely unchanged over the past 20 years; additionally, there is no proven second-line regimen for poor responders. The five-year survival rate remains at best 70% and it is much lower for patients who present with or progress to metastatic disease^3–7^. While recurrent germline and somatic mutations (including in *RB1, TP53*, and *ATRX*)^8–15^ and unbalanced chromosomal rearrangements (including allele loss on 3q, 13q, 17p, and 18q)^16,17^, have been identified, there is no characteristic OSA translocation and significant genetic heterogeneity exists, posing a challenge with respect to diagnosis and identification of treatment targets. Furthermore, clinical assessment of chemotherapy response and identification of patients who would benefit from additional-line agents is currently reliant primarily on histologic tumor necrosis^6,18^, an imperfect surrogate which can only be assessed after multiple rounds of therapy have been administered^19–21^. Recently, when pathologically assessed response to chemotherapy was used as a marker to stratify patients for alternate or intensified adjuvant therapies in a large international clinical trial (EURAMOS), it did not result in a survival benefit^6,22,23^. This failure underscores the need for improved prognostic biomarkers to assist in the development of therapies.

Recent work has focused on post-translational modifications and epigenetic alterations as potential prognostic markers and therapeutic targets. Our group has demonstrated that microRNA (miRNA) expression patterns from various genomic loci, predominantly those from the 14q32 non-coding region, are predictive of clinical outcome and capture prognostic information distinct from that conveyed by pathologic necrosis and/or metastatic status^24–27^. We have also previously examined the modulation of miRNA expression by DNA methylation at 14q32^25,27^, but the applicability of *global* methylation patterns in OSA remains much less clear.

Induction of global hypomethylation has been demonstrated to result in chromosomal instability and OSA formation in animal models^28^, and in vitro treatment with demethylating agents has been shown to reverse epigenetically silenced tumor suppressor genes and inhibit OSA cell growth^29–31^. These observations are consistent with data showing that DNA methylation inhibitors preferentially affect genes that are expressed in normal tissue and silenced in cancer^32–34^. Genome-wide methylation signatures in clinical samples have been utilized to distinguish OSA from Ewing or synovial sarcoma, underscoring the notion of epigenetically-modified molecular phenotypes^35^, and global methylation patterns have been shown to be prognostic of relapse risk in a small OSA clinical cohort^36^.

In a rare and mostly pediatric tumor, like OSA, one limiting factor is the lack of easily accessible large and well-annotated specimen cohorts. In this respect, we leveraged the large NCI TARGET dataset (Therapeutically Applicable Research to Generate Effective Treatments), which is a clinically annotated, multi-omic dataset, recently released by the NCI in order to facilitate genomic research in OSA. We perform extensive bioinformatic analysis of the TARGET data and present analyses of genome-wide (and CpG site subtype-specific) methylation patterns identifying methylation profiles associated with clinical outcomes. We validate the clinical prognostic and predictive value of our molecular profiles in the only other public OSA methylation profiling dataset with sample survival information currently available, and the reproducibility of the molecular patterns in two additional independent datasets. Finally, making use of in vitro cell line and pharmacogenomic data, we identify candidate gene targets and drugs. In conclusion, we propose DNA methylation-based subtypes that warrant further exploration as a prognostic tool for patient risk stratification for therapies and tailored therapeutic targeting and drug repurposing in OSA.

## Results

### Genome-wide methylation patterns of primary OSA samples are associated with clinical outcomes

We identified four clinical datasets collectively composed of 147 samples. Three datasets were analyzed by the same genome-wide array-based methylation assay (Illumina Infinium HumanMethylation450 BeadChip (450K array)) and the fourth used a HELP-tagging assay. Clinical characteristics of the four cohorts, TARGET (*n* = 83), Albert Einstein College of Medicine^36^ (AECM, *n* = 15), Japanese National Cancer Center Research Institute^30^ (JNCCRI, *n* = 34), and New York^35^ (NY, *n* = 15), are presented in Supplementary Data 1. 141 samples are biopsies or diagnostic resections collected prior to therapy. Six metastatic samples were also included in the JNCCRI dataset. The TARGET initiative sought to characterize pediatric cases, so it does not include older adult cases found in the other datasets. The JNCCRI and NY datasets included a larger fraction of axial cases. Available information indicates most patients were treated with standard chemotherapy (methotrexate, Adriamycin (doxorubicin), and cisplatin (MAP)), although treatment information is not available for all patients.

**Fig. 1:**
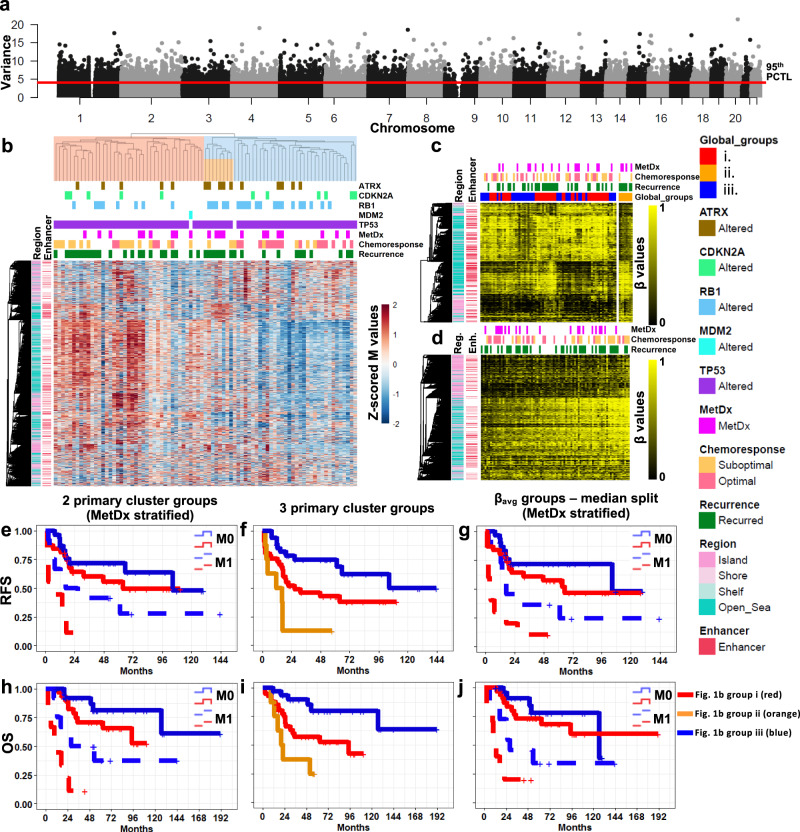
Global methylation patterns of primary tumors and clinical associations. **a** Manhattan plot of genome-wide variance in methylation. The 5% most variantly methylated sites, those above the red line, are analyzed as the Global profile. **b** Unsupervised hierarchical clustering of the TARGET samples using the Global profile. Cluster reproducibility (R) indices were high, 0.98/0.95 for two and three groups respectively. **c** Supervised β value heatmap of the 1725 CpG sites differentially methylated (FDR < 0.1) between the very poor prognosis (ii) and other cluster groups (i and iii) in (**b**). **d** Supervised heatmap of the Global profile. Samples are ordered from low to high average β value. Two sample groups generated by a median split of the average β values were significantly associated with pathologic response to chemotherapy (Fisher’s exact *p* = 0.002, OR = 10.2, 95% CI: 2.5–41.7). **e** MetDx stratified RFS analysis of the two primary Global cluster groups (not metastatic: median RFS = 63.5 (i) and 104.7 mo. (iii), metastatic: median RFS = 2.3 (i) and 26.7 mo. (iii), pooled Log-Rank *p* = 0.006). **f** RFS analysis of the three primary cluster groups (median RFS = 11.5 (ii), 26.7 (i), and 104.7 mo. (iii), Log-Rank *p* = 5.0 × 10^−4^). Pairwise RFS analysis of the three primary cluster groups. Short (ii, 11.5 mo.) vs. intermediate (i, 26.7 mo.) median RFS Log-Rank *p* = 0.034. Intermediate (i) vs. long (iii, 104.7 mo.) median RFS Log-Rank *p* = 0.022. Short (ii) vs. long (iii) median RFS Log-Rank *p* = 2.7 × 10^−5^. **g** MetDx stratified RFS analysis of the low and high average methylation groups (median split) from **c** (not metastatic: median RFS = 65.5 and 104.7 mo., metastatic: median RFS = 3.5 and 16.9 mo., pooled Log-Rank *p* = 0.044). **h** MetDx stratified OS analysis of the two primary cluster groups (not metastatic: median OS = NYR (i) and NYR (iii), metastatic: median OS = 20.6 (i) and 111.9 mo. (iii), pooled Log-Rank *p* = 5 × 10^−4^). **i** OS analys**i**s of the three primary cluster groups (median OS = 20.4 (ii), 94,7 mo. (i), and NYR (iii), Log-Rank *p* = 7.4 × 10^−4^. Pairwise OS analysis of the three primary cluster groups. Short (ii, 20.4 mo.) vs. intermediate (i, 94.7 mo.) median OS Log-Rank p = 0.067. Intermediate (i) vs. long (iii, NYR) median OS Log-Rank *p* = 0.008. Short (ii) vs. long (iii) median OS Log-Rank *p* = 1.8 × 10^−4^. **j** MetDx stratified OS analysis of the low and high average methylation groups (median split) from (**c**) (not metastatic: median OS = 126.6 mo. and NYR, metastatic: median OS = 9.7 and 28.2 mo., pooled Log-Rank *p* = 0.076).

We first sought to discover if genome-wide methylation patterns offer insight into OSA clinical outcomes using the large, well-annotated NCI TARGET dataset. Methylation β values were downloaded from the TARGET data matrix. Standard pre-processing including out-of-band signal intensity correction, Lumi dye bias color correction, and beta mixture quantile dilation probe bias normalization was already applied. β values were converted to M values for statistical testing. We considered the 5% most variant CpG methylation sites (based on M values) in the dataset to remove statistical noise, leaving 19,264 sites for analysis (Fig. 1a, Supplementary Data 2). We refer to these sites as the Global profile. The Global profile was highly enriched (hypergeometric *p* ≪ 0.05) for sites in CpG Islands (CGIs) not associated with gene promoters, and Open Sea regions, parts of the genome with low CpG density when compared all sites interrogated by the array (Supplementary Table 1). Additionally, there was a large enrichment for intergenic CpG sites not annotated to any known gene (*p* = 1.03 × 10^−281^).

Unsupervised hierarchical clustering using the Global profile revealed two patient subgroups with strikingly different methylation patterns where one subgroup was largely hypermethylated relative to the other (Fig. 1b). The hypermethylated subgroup was enriched for tumors unresponsive to standard chemotherapy (pathologically assessed tumor necrosis in response to pre-operative chemotherapy (chemoresponse) < 90%, Odds ratio (OR) = 6.429, 95% CI = 1.662–24.860, Fisher’s Exact Test (2-sided), *p* = 0.007). The hypermethylated group displayed a trend for shorter Recurrence Free Survival (RFS) and Overall Survival (OS) in Kaplan–Meier analysis (Supplementary Fig. 1), and were significantly associated with both outcomes when we stratified patients by the presence of metastasis at diagnosis (*p* = 0.006, *p* = 5 × 10^−4^, Fig. 1e, h). The two main cluster groups were not different with respect to age (*p* = 0.531), metastasis at the time of diagnoses (MetDx, a known strong prognostic factor, *p* = 0.615), or sex (*p* = 0.661). We tested differential methylation between the two groups, and found many strong associations (11,881 sites, *p* < 0.001, Benjamini–Hochberg False Discovery Rate (FDR)^37^ < 0.1), 46.8% of which had an average β value difference > 0.2. 98.5% of these sites were hypermethylated in the poor prognosis cluster subgroup (Fig. 1b, red). Large average β value differences (∆β > 0.4) were found at 0.7% of sites, all of which were hypermethylated in the poor prognosis cluster subgroup. Open Sea and intergenic sites were enriched in the differentially methylated sites compared to 5% most variant sites (Supplementary Table 1). There was a high prevalence of sites located in the chromosome 14q32.2–32.31 locus among the top sites. Nineteen of the 100 most differentially methylated sites were located here, including the most differentially methylated site, cg08175935 which is annotated to *MIR127*, *MIR433*, and *RTL1* (Supplementary Data 2). 14q32 is an imprinted region encoding many microRNAs^38,39^, the transcription and methylation status of which we have previously reported being prognostic of patient survival^24,25,27^. Region-based analysis comparing the cluster groups performed using *minfi*^40^ and *bumphunter*^41^ revealed 60 regions differentially methylated (family-wise error rate < 0.1), 59 of which were hypermethylated in the poor prognosis group (Supplementary Data 3). Ten of these regions were in 14q32 non-coding cluster. Eight other regions also overlapped known imprinted loci, including three in the tumor suppressor region of chromosome 11p15 associated with Beckwidth–Weidman syndrome^42^. Fifteen regions on chromosome 6 encompassing the HLA locus were identified, as well as five additional regions about 0.5 MB telomeric of the locus overlapping tRNAs and replication-dependent histones. While not imprinted, many genes in the HLA locus exhibit monoallelic expression^43^.

When we considered three, instead of two, main clustering groups, we noticed an increased RFS and OS discrimination (Fig. 1f, i). These three groups remained significantly associated with both outcomes when stratified for metastasis at the time of diagnosis (Supplementary Fig. 1). The third subgroup was a subset of the hypomethylated subgroup but displayed more balanced methylation pattens and its members had poor long-term survival. The only two samples in this subgroup with chemotherapy information had suboptimal responses, and half of the patients in this subgroup presented with metastatic disease. We compared the methylation profiles of the small cluster group with exceptionally poor prognosis to the other samples and identified 1725 differentially methylated sites (FDR < 0.1, Fig. 1c), 92.4% of which had an average β difference > 0.2, and 48.9% which were hypermethylated in the poor prognosis group. 8.3% of the sites had an average β difference > 0.4, of which 48.6% were hypermethylated in the poor prognosis subgroup. The differentially methylated sites were strongly enriched in Open Sea and Enhancer regions (Supplementary Table 1). Inspection of the β values at these sites revealed the poor prognosis group is hypermethylated at a small number of CGIs and a subset of Open Sea sites, and hypomethylated at a larger subset of Open Sea sites (Fig. 1c).

We also sought to explore individualized patient outcome prediction using the global methylation patterns by applying a supervised average methylation approach. To do this, we ranked patients by their average β value of the Global profile and created two, three, and four patient groups using the median, tertile, and quartile average methylation values. These patient groups were significantly associated with response to chemotherapy (χ^2^ test, *p* = 0.002, 0.002, and 0.017, respectively, Fig. 1d). We observed an inverse relationship between global methylation and chemoresponse where hypomethylated samples were more likely to have an optimal response and hypermethylated samples were more likely to have a suboptimal response. Furthermore, we found evidence of a methylation stratified effect between methylation and chemoresponse when we performed pairwise comparisons between each of the groups, as all the odds ratios displayed a consistent and expected relationship (OR < 1). A trend for association between these same β value-based risk groups and RFS was observed (Supplementary Fig. 1), and the groups were significantly associated with RFS and trending for association with OS when stratified for metastatic disease at diagnosis (Fig. 1g, j).

**Fig. 2:**
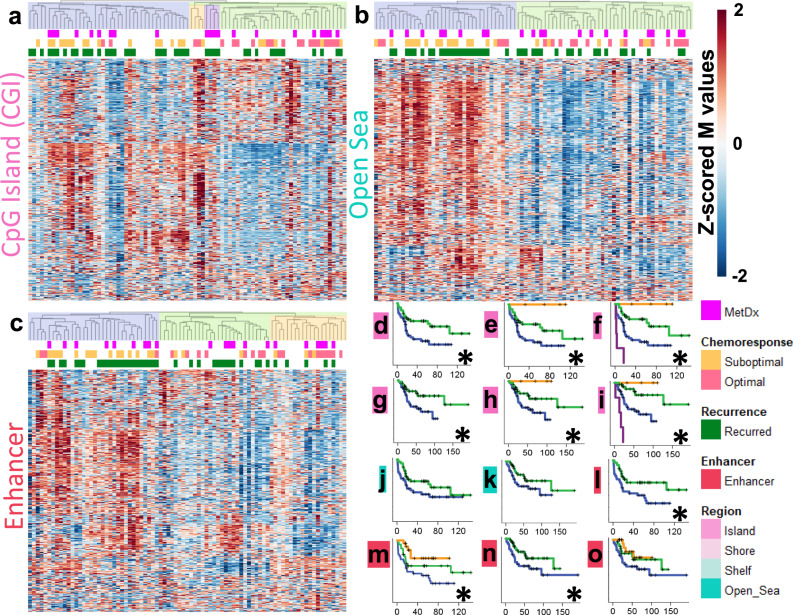
Methylation patterns of genomic regions. **a** Unsupervised hierarchical cluster analysis using CGI methylation (R indices = 0.83, 0.92, and 0.80 for 2, 3, and 4 groups, respectively). **b** Cluster analysis using Open Sea methylation (R index = 0.72 for 2 groups). **c** Cluster analysis using Enhancer methylation (R index = 0.80/0.76 for 2 and 3 groups respectively). **d**–**f** RFS analysis of the 2, 3, and 4 primary CGI clusters (Log-Rank *p* = 0.009, 0.015, and 6.7 × 10^−7^, respectively). **g**–**i** OS analysis of the 2, 3, and 4 primary CGI clusters (Log-Rank *p* = 0.016, 0.036, and 3.2 × 10^−8^, respectively). **j** RFS analysis of the 2 primary Open Sea clusters (Log-Rank *p* = 0.074). **k** OS analysis of the 2 primary Open Sea clusters (Log-Rank *p* = 0.084). **l**, **m** RFS analysis of the 2 and 3 primary Enhancer clusters (Log-Rank *p* = 0.008, and 0.015, respectively). **n**, **o** OS analysis of the 2 and 3 primary Enhancer clusters (Log-Rank *p* = 0.045, and 0.116, respectively). Significant survival differences (log-rank *p* < 0.05 are marked with an *).

We then investigated if methylation patterns of specific genomic regions differ from those observed at the genome-wide scale and if they offer additional insights into clinical outcomes. We thus performed hierarchical clustering using CGI, Shore, Shelf, Open Sea, and Enhancer regions. The two main cluster groups generated using methylation of each of these regions were not associated with patient age (Fisher’s exact test, *p* > 0.05). CGI-defined cluster groups exhibited a balance of relative hyper and hypomethylation (Fig. 2a), contrasting the patterns observed across the entire genome (Fig. 1b), and in CpG sparse regions (Open Sea, Fig. 2b). The methylation patterns of each region (except for Shelf regions) generated two groups of samples with very different responses to chemotherapy (Fisher’s exact test, OR > 8, *p* < 0.005 for all four analyses). The suboptimal response subgroup was largely hypermethylated in the Shore, Open Sea, and Enhancer regions. Sample risk subgroups generated by CGI and Enhancer methylation patterns provided the strongest discrimination for RFS and OS (Fig. 2d–i, l–o, significant (KM log-rank *p* < 0.05) results marked with *). When we stratified for MetDx, we observed that the cluster groups generated with each of the genomic regions were significantly (KM log-rank *p* < 0.05) associated with RFS and OS (Supplementary Fig. 2). Average β value-based risk groups generated using only CGI or Enhancer sites were not associated with survival, unlike groups generated by clustering, given the mix of hyper and hypomethylation in most samples observed in Fig. 2. However, average β value-based risk groups generated with each region were significantly associated with chemotherapy response (*p* < 0.05) and the hypermethylated groups contained fewer responders.

### Methylation signatures are associated with clinical outcomes in univariate probe-level analysis

**Fig. 3:**
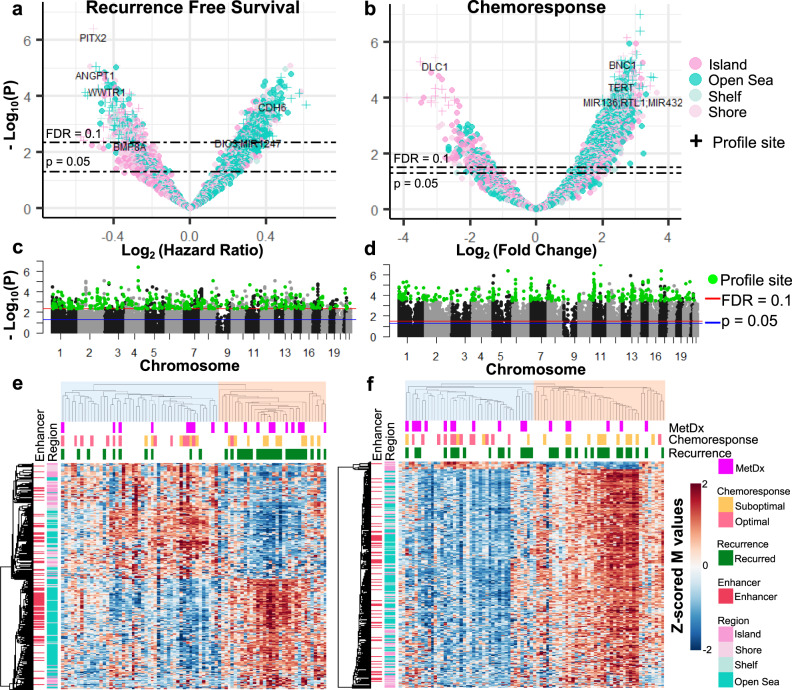
Supervised methylation profiles associated with outcome. **a**, **b** Volcano plots for the association between methylation and RFS and CR. **c**, **d** Manhattan plots for association between methylation and RFS and CR. RFS and CR profile sites are in green. **e**, **f** Semi-supervised hierarchical clustering using the RFS and CR profiles (2-group cluster R-indices = 0.846 and 0.862, respectively).

After observing that broad methylation patterns are associated with outcome, we sought to discover subsets of individual CpG methylation sites most strongly associated with outcome and thus potentially clinically applicable for prognostication. We analyzed individual probes for association with RFS and CR using a Benjamini-Hochberg FDR corrected *p* value of 0.1 as a cutoff to identify sites associated with the RFS and CR outcomes. With this approach, we found 885 sites were associated with RFS and 6224 associated with CR (Fig. 3a–d and Supplementary Data 2). Additionally, we tested for associations with MetDx, but did not identify any significantly associated sites. We observed a significant degree of overlap between the RFS and CR associated lists (hypergeometric test *p* < 0.001), so we refined more specific outcome profiles by removing probes shared by more than one of these lists. This generated a list of 374 sites associated with RFS and 5641 with CR. We trimmed the CR profile to the 374 most significant sites to increase specificity and match the size of the RFS profile. Both the RFS and CR profiles were significantly enriched (hypergeometric test *p* < 0.05) for sites in CpG sparse regions (Open Sea) and depleted for sites in CpG dense regions (CGI). The depletion of CGI-associated sites was strongest for non-promoter CGIs in the RFS profile, and promoter-associated CGIs in the CR profile. Additionally, the RFS profile was significantly enriched for intragenic sites, whereas the CR profile was significantly enriched for intergenic sites (complete results in Supplementary Table 1).

We secondarily tested for methylation associations with the more distant OS endpoint and identified 149 sites significantly associated (FDR < 0.1) with this outcome, 93.3 and 29.5% of which were members of the 885 and 374 RFS associated site lists, respectively. In a targeted manner, we tested the RFS-associated sites for association with OS. Not surprisingly, we found 97.6 and 95.7 % of the 885 and 374 RFS sites were also significantly associated (FDR < 0.1) with OS.

Then using the refined outcome profiles, we performed semi-supervised hierarchical clustering to visualize the methylation patterns (Fig. 3c, d). The two primary patient cluster groups were not different with respect to age or sex (Fisher’s exact test *p* > 0.05). The RFS profile displayed a balance of hypo and hypermethylation across patients, where the poor prognosis subgroup (red) was defined by predominantly hypomethylated CGIs and hypermethylated Open Sea genomic regions (Fig. 3e). We observed that the RFS signature predicted a few patients with suboptimal chemotherapy response to achieve long-term survival, noteworthy as it is known that a small subset of patients who do not respond to therapy can still have good outcomes. The CR profile displayed methylation patterns similar to the Global profile (Figs. 1b, 3f), with a largely hypermethylated poor chemoresponse group and a hypomethylated optimal chemoresponse group. Of the small subset (*n* = 13) of CR profile CpG sites with an opposite methylation-chemoresponse association, 11 were in CpG islands, and the other two were in neighboring Shore regions.

We tested if the outcome profiles produced similar sample classifications to the Global profile. The CR profile generated sample classifications highly concordant with the Global profile (Cramer’s *V* = 0.783, Fisher’s exact test *p* = 1.18 × 10^−13^). The RFS profile also classified samples similar to the Global profile (Cramer’s *V* = 0.255, *p* = 0.026), although the association was not as strong, indicating long-term outcome and tumor aggressiveness may be mediated by different and more specific epigenetic mechanisms than response to chemotherapy.

While we avoided testing potential associations between the outcome defined methylation signatures and their respective outcome out of concern for overfitting, to illustrate the effect size of the constituent CpG sites in the RFS profile, we performed KM analysis using patient groups defined by methylation value quantiles of select sites of possible biologic interest ranked close to the top of list (Supplementary Fig. 3). We observed a potential methylation level stratified relationship between methylation of these sites (and others) with RFS, whereby more extreme levels of methylation were associated with more extreme survival times.

### OSA methylation profiles are different from CRC and glioma CpG island methylator phenotypes

Methylation profiles, specifically CpG island methylator phenotypes (CIMPs), have been described in malignancies including colorectal cancer and glioma. CIMP positive tumors are defined by their aberrant hypermethylation of CGIs, frequently have characteristics mutations (eg *BRAFV600E* in CRC and *IDH* in glioma), and may have unique clinical characteristics^44–46^. Thus, we tested if OSA methylation patterns were associated with the genomic alterations reported in the TARGET dataset, namely *ATRX*, *CDKN2A*, *MDM2*, *RB1*, and *TP53*. Comparing the two primary Global profile patient subgroups (Fig. 1b), we found that *RB1* alterations were enriched in the hypomethylated (good prognosis) subgroup (Fisher’s *p* = 0.021, OR = 3.18, 95% CI = 1.134–9.547, Supplementary Table 2). *RB1* interacts with epigenetic modifiers such as histone demethylases^47^ and *RB* knockout has been associated with hypomethylation and genomic instability in mouse embryonic fibroblasts. However, the imperfect clustering of *RB1* mutants suggests that factors beyond *RB1* are involved in the prognostic methylation patterns. Additionally, we found that the small, intermediately methylated subgroup with extremely poor prognosis was enriched for *ATRX* alterations (*p* = 0.003). *ATRX* plays a role in epigenetic regulation^48^, holds prognostic value in other pediatric tumors^49^, and there are ongoing efforts to develop *ATRX* inhibitors for the treatment of sarcomas, including osteosarcoma. The imperfect, but relatively tight clustering of *ATRX* mutants raises the possibility of tailored therapeutic application of demethylating agents with *ATRX* inhibitors. We did not find genomic alteration enrichment in either the RFS or CR profile-based cluster groups (Fig. 3c, d), suggesting these methylation profiles convey predictive/prognostic information independent of known recurrent genomic alterations in OSA.

We also tested if the OSA methylation patterns resembled CRC and glioma CIMPs using gene panels reported by Toyota et al.^44^ (“Toyota panel”), Weisenberger et al.^45^ (“Weisenberger panel”) for CRC, and Noushmehr et al.^46^ (“Noushmehr panel”) by generating “CIMP Scores” for each sample in the TARGET dataset based on the average β value of panel gene associated sites (Supplementary Data 1). Two scores were generated for each sample with each panel, one using all CGI sites annotated to the panel and another using the subset of sites in promoter regions. CIMP scores were compared between the profiles’ main cluster groups. While at least one panel’s CIMP score was significantly different between each profile’s cluster groups (t-test, *p* < 0.05, Supplementary Table 3), absolute differences in methylation were small (∆β_avg_ < 0.05) and the scores were positively correlated (Spearman, *p* = 0.01–<1 × 10^−9^) with the Global average methylation level, suggesting these associations may be surrogates for the larger genome-wide differences. CIMP scores were not prognostic by Cox regression (*p* > 0.05), even when patients were stratified for M status, and did not contribute prognostic power to a Cox model generated using the Global cluster groups (omnibus test of model coefficients *p* > 0.05). Clustering using the CIMP panels did not reveal the striking methylation patterns and clearly defined groups reported in CRC and glioma (Supplementary Fig. 4). The Weisenberger panel promoter-associated sites revealed slight hypermethylation in a small subset of samples, however, this relationship was not observed with the Toyota panel, which measures the same CRC CIMP phenotype, suggesting the OSA methylation patterns may be distinct from CRC CIMP.

We also tested if the CIMP scores were associated with the same gene alterations reported in the TARGET dataset (Supplementary Table 3). The Noushmehr and Weisenberger panel CIMP Scores were found to be greater in *CDKN2A* altered tumors (*p* = 0.003 and 2 × 10^−4^). The *CDKN2A* mutants clustered imperfectly when using the panels and these associations are weaker than the very strong correlations between *IDH* and *BRAFV600E* mutations and CIMP status in glioma and CRC, respectively. The methylation signal in OSA appears to be different from those in CRC and glioma, especially considering the OSA profiles are enriched for Open Sea and intergenic sites. While in our analysis the CGI-based patient cluster groups (Fig. 2a) were the most significantly associated with RFS of all genomic regions, there was not a consistent hyper vs hypomethylated pattern differentiating the groups, unlike the reported CIMP phenotypes.

### Methylation patterns are predictive of RFS independent of known prognostic factors

Pathologic necrosis following pre-operative chemotherapy is the single validated prognostic factor in OSA used to stratify patients for new therapies. Given the observation that there was discordance between chemoresponse and the patient risk groups classified by the RFS profile, we tested if the methylation markers predicted survival independent of chemoresponse, with the understanding that this result will need additional validation when data becomes available given that the RFS profile was defined in the TARGET dataset, and thus could be expected to overperform. We observed that 15.2% and 6.7% of the RFS profile and all CpG sites found associated with RFS, respectively remained significantly predictive (FDR < 0.1) of RFS over and above chemoresponse in a multivariate Cox regression, despite the analysis being performed on the smaller subset of samples (*n* = 42) with chemoresponse information (Supplementary Data 2). We analyzed the representation of genomic regions in the subset of the RFS profile predictive of RFS independent of chemoresponse and found that CGI’s were enriched (hypergeometric test, *p* = 0.003) and Open Sea regions were depleted (*p* = 0.005) relative to the complete profile.

Our group, and others, have also proposed that miRNA transcriptional profiles could be a useful prognostic marker for this disease^24,25,27^. Therefore, we used the microRNA assays that were reported for the same specimens in the TARGET dataset to perform another multivariate analysis which demonstrated 100% of both the RFS profile and all CpG sites found associated with RFS were predictive of RFS over and above miRNA transcriptional risk scores (Supplementary Data 2).

### Methylation profiles are reproducible in independent datasets

We next examined the AECM dataset^36^, currently the only other publicly available OSA methylation dataset with patient survival information. Overall, 8.1, 8.6, and 11.5% of the sites in the Global, RFS, and CR profiles defined using TARGET 450k array data mapped to the HELP-tagging assay^50^. Additionally, 8.9 and 8.7% of all sites associated with RFS and CR were mapped. Despite the small sample size (*N* = 15), the very different methylation assay used, and a high degree of mapping attrition, we still sought to validate our main findings from the TARGET dataset. Given the difference in assay technology, supervised models could not be used for outcome prediction, so we performed hierarchical clustering using the profiles and tested the resulting sample groups for association with clinical outcomes.

**Fig. 4:**
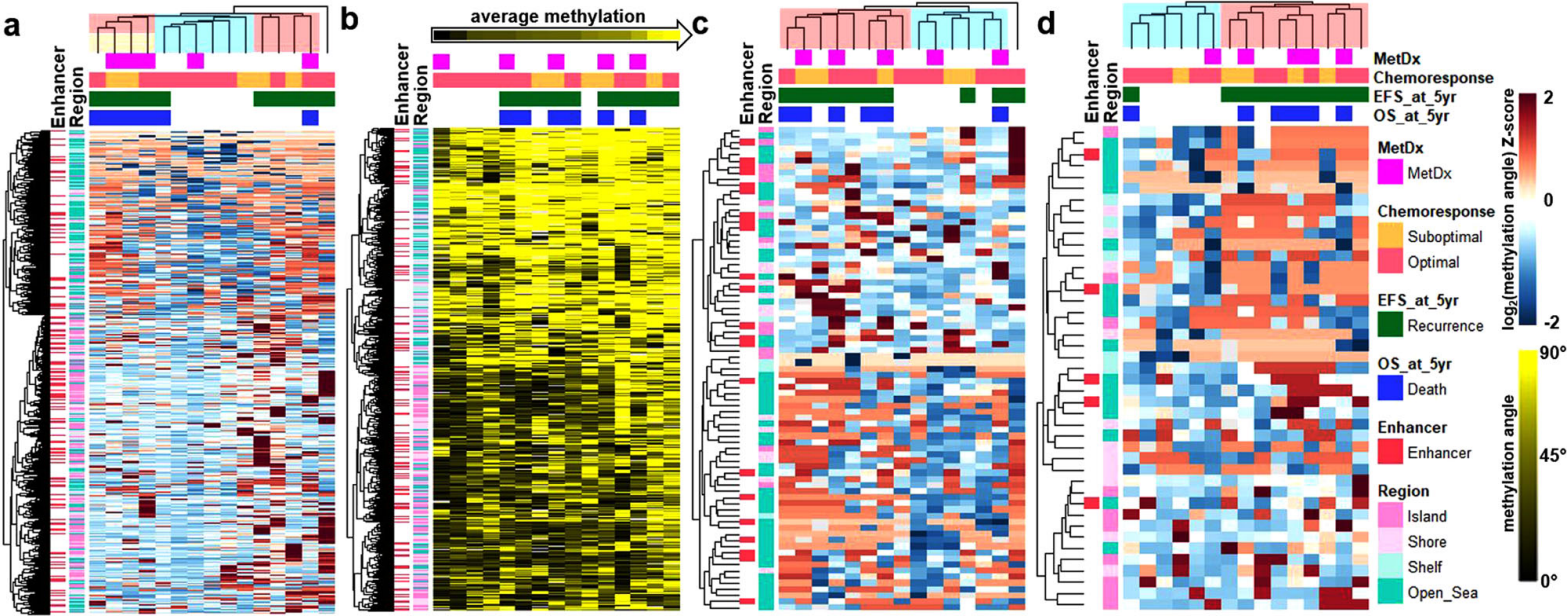
Methylation profiles in the AECM validation dataset. **a** Hierarchical clustering using the Global profile. **b** Supervised heatmap of the Global profile. Samples are ordered from low to high average methylation value. **c** Hierarchical clustering using sites associated with RFS in the TARGET dataset. **d** Hierarchical clustering using the CR profile.

Unsupervised hierarchical clustering using the Global profile revealed three groups, one of which was largely hypomethylated (blue) relative to the other two groups (red), similar to the findings in the TARGET dataset (Fig. 4a). The hypomethylated group had significantly better Event Free Survival (EFS, binarized at 5 years) than the hypermethylated groups (Fisher’s *p* = 0.003), contained all five patients that did not experience an event before 5 years, and five of the six samples from patients alive after 5 years. Additionally, the hypermethylated groups contained four of the five samples that did not respond to chemotherapy. Considering three groups individually, we noticed that in addition to the hypomethylated group with good outcomes the leftmost cluster group with a balance of hyper and hypomethylation (orange) had an extremely poor prognosis, with none of the four patients alive after five years (*p* = 0.015).

We then supervised rank-ordered samples by average Global profile methylation level (Fig. 4b). The quartile with the lowest methylation all had an optimal response to chemotherapy and did not have a recurrence or death before 5 years (*p* = 0.003). Conversely, of the quartile with the highest methylation three died before 5 years, and all had a recurrence before 5 years. By median split, the highly methylated samples had an Odds Ratio of 9.3 for an event at 5 years (*p* = 0.119) and included four of five samples with suboptimal chemoresponse.

Hierarchical clustering using the sites that were associated with RFS in the NCI TARGET dataset, revealed two sample groups, one largely hypermethylated relative to the other (Fig. 4c). The pattern of relative hyper/hypomethylation of these sites was more balanced than in the TARGET dataset. Like the TARGET dataset, one of the two main methylation site cluster groups was mostly composed of Open Sea sites, and these sites were hypomethylated in patients with a trend for good prognosis (five-year EFS *p* = 0.091, five-year OS *p* = 0.138). Additionally, when the more specific RFS signature (with only 32 of 374 CpG sites (8.6%) mapped in the dataset), was used to cluster samples, the two primary groups had significantly different event rates at five years (*p* = 0.022), though with an imbalanced sample grouping (Supplementary Fig. 5).

Hierarchical clustering using the CR profile revealed two main groups of samples, one largely hypermethylated relative to the other, and four of the five samples with suboptimal chemoresponse clustered in the hypermethylated subgroup (Fig. 4d).

**Fig. 5:**
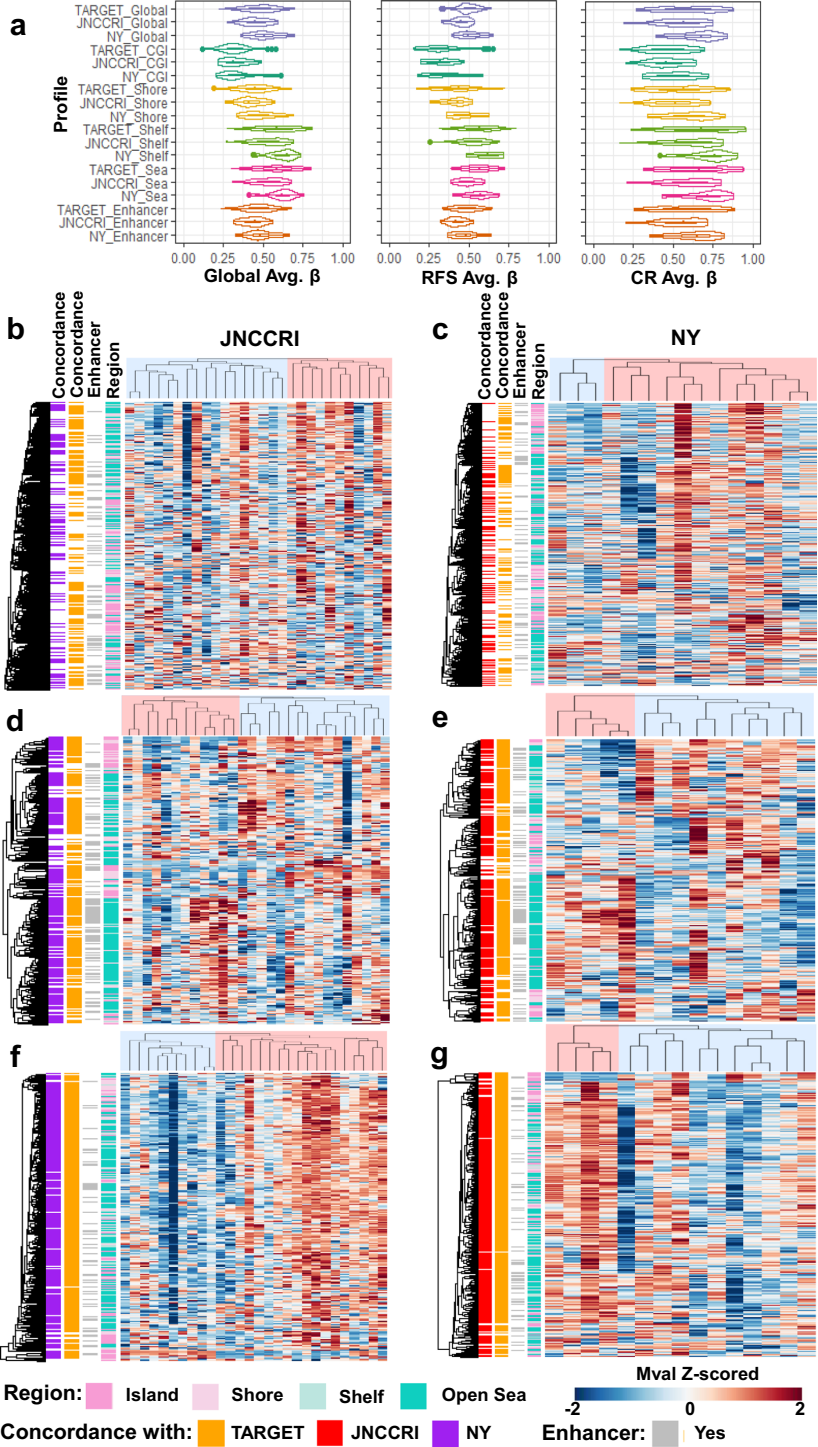
Methylation profiles in two independent 450k array OSA datasets. **a** Average β value distributions of Global, RFS, and CR profiles and region-specific subsets. Semi-supervised hierarchical clustering of the Global profile (**b**, **c**), RFS profile (**d**, **e**), and CR profile (**f**, **g**) in the JNNCRI (left) and NY (right) datasets. CpG sites displaying concordant hypo/hyper-methylation patterns between the independent 450k array datasets are annotated in the first two row annotation tracks of each heatmap. Detailed concordance and differentia methylation results between the cluster groups are presented in Supplementary Table 4.

We also analyzed two additional independent publicly available datasets (JNCCRI^30^ and NY^35^) generated using the Illumina 450k array to further test if we could reproduce the methylation patterns discovered in the TARGET dataset. Chemoresponse and survival data were not available in these public cohorts, so we could not directly test the prognostic and predictive value of our signatures. We instead investigated if the profiles classify samples similarly and displayed similar methylation patterns to those observed in the TARGET dataset. We first observed the distribution of the signatures’ average β values across samples (Fig. 5a) and found them to be similar by broad inspection, albeit with a trend towards hypermethylation in the NY dataset. We performed semi-supervised hierarchical clustering using the profiles in the independent datasets and analyzed the methylation patterns of the two primary cluster groups in each analysis (Fig. 5b–g). In both independent datasets, the Global and CR profiles were almost entirely hypomethylated in one group relative to the other, and the RFS profile displayed hypo and hypermethylation in each risk group, as was observed in the TARGET dataset. Sample subgroups generated with each of the profiles in each dataset were not different with respect to age (Fisher’s exact test *p* > 0.05), except for those generated using the RFS profile in the JNCCRI dataset (*p* = 0.009).

We performed differential methylation analysis between the two primary cluster subgroups in each dataset and calculated the fraction of CpG sites which had concordant fold change directions between the datasets using all of the profile sites, and those differentially methylated (FDR < 0.1) between the cluster groups being compared (Fig. 5b–g and Supplementary Table 4). The methylation patterns in the JNCCRI and NY datasets were remarkably similar to those observed in the TARGET dataset. This was especially true for the RFS and CR profiles.

**Fig. 6:**
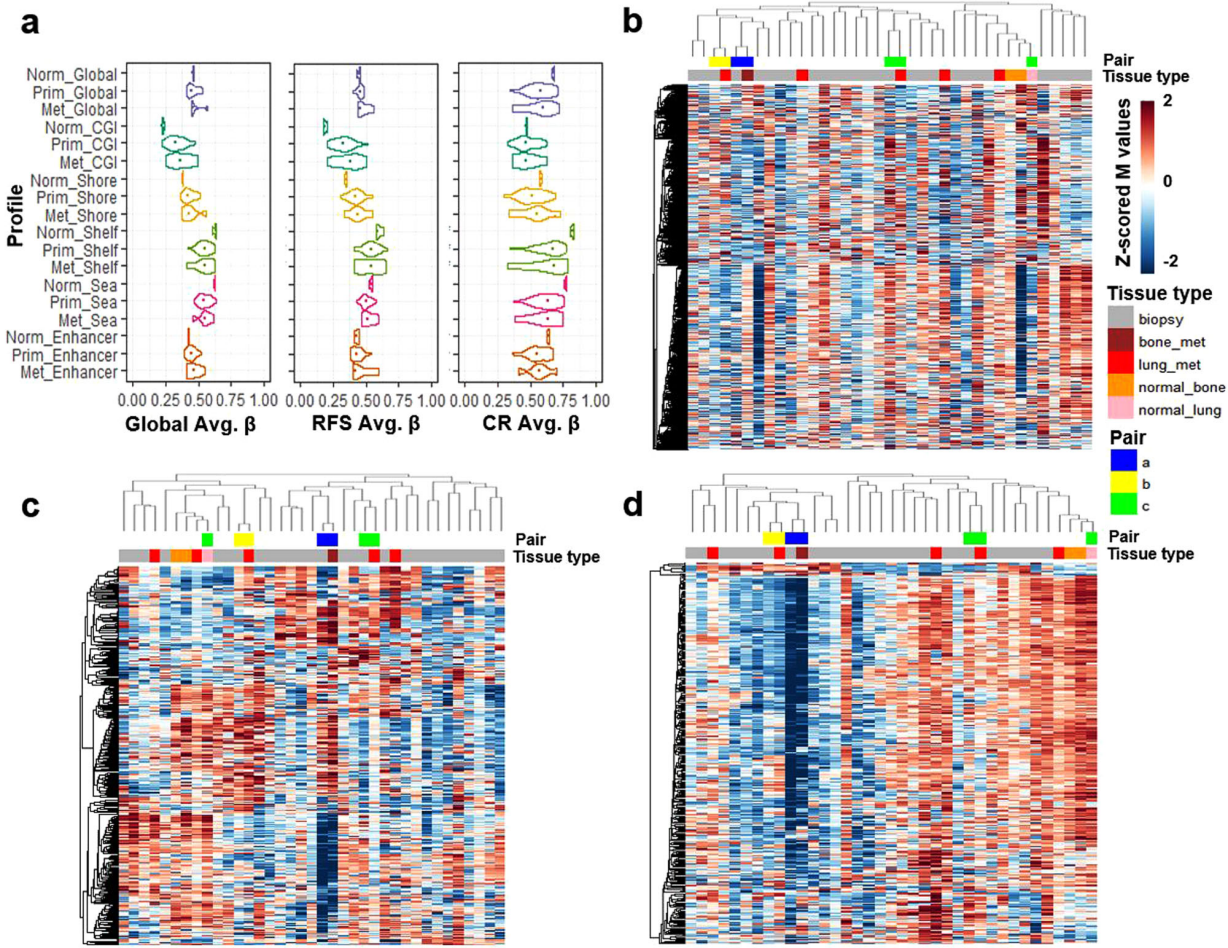
Methylation profiles in the JNCCRI dataset including primary as well as metastatic tumor and normal tissue. **a** Average β value distributions of Global, RFS, and CR profiles and region-specific subsets. Semi-supervised hierarchical clustering of normal, primary, and metastatic samples using the Global (**b**), RFS (**c**), and CR (**d**) profiles.

The JNCCRI dataset contained unique information for two normal bone, one lung, and six metastatic samples, which, although of limited sample size and heterogeneous sources, offered some additional insights. We observed that the average methylation level of CGIs was elevated, and the average methylation level of Open Sea regions was depressed in tumor compared to normal tissue, a relationship commonly observed in other cancers (Fig. 6a)^51^. We also found Shore patterns mirrored those of CGIs, and Shelf patterns mirrored those of the Open Sea regions. While most CGI’s are hypomethylated in normal tissue^52^, the CGIs in the CR profile were found to be relatively hypermethylated in normal tissue. Furthermore, the CR profile, in general, was relatively hypermethylated in normal tissue, suggesting that tumors with more normal-like methylation patterns are less likely to respond to therapy. The RFS profile was largely hypermethylated in tumors, the CR signature was hypomethylated in tumors, indicating the profiles may be tracking distinct elements of biology. We also performed semi-supervised hierarchical clustering using the outcome signatures with normal, primary, and metastatic samples (Fig. 6b–d). Normal tissue samples always clustered together, and the tumors predicted to be more aggressive/less response to therapy. Metastatic samples did not cluster together, however primary-metastatic tissue pairs were always grouped as more similar to each other than any other sample. This raises the possibility that the profiles may not substantially change with metastatic tumor progression, though this should be validated in future larger dedicated cohorts.

### Methylation offers insight into potential targeted application of immune checkpoint inhibitors

CpG sites from a previously reported epigenetic signature (EPIMMUNE)^53^ generated using another Illumina methylation platform (EPIC array) for response to the immune checkpoint inhibitors nivolumab and pembrolizumab in non-small cell lung cancer were enriched in the TARGET dataset’s most variant sites (hypergeometric test, *p* = 1.94 × 10^−14^). The EPIMMUNE signature contains 301 CpG sites, 128 of which were interrogated by the lower resolution 450k array and passed the pre-processing criteria in the TARGET dataset. These sites were not enriched in the outcome signatures, but we did find through differential methylation analysis that 34 of them were univariately associated with response to chemotherapy (FDR < 0.1). Additionally, hierarchical clustering of the TARGET samples using the EPIMMUNE signature revealed that a subset of OSA samples had methylation patterns similar to the lung cancer samples that responded to immunotherapy (Supplementary Fig. 6). Notably, these samples belong to the hypermethylated, poor chemoresponse Global profile group (Fig. 1b, red).

We then examined if the observed methylation profiles were associated with immune infiltration. Percent cellular tumor content reported by the TARGET investigators was not significantly different between the main cluster groups derived by the Global, RFS, or CR profiles, but was significantly lower in the EPIMMUNE derived cluster group containing the samples with methylation patterns associated with good response to PDL1 based immunotherapy (*p* = 0.005, Supplementary Table 5). ESTIMATE^54^ and CIBERSORTx^55,56^ were used to evaluate specific immune cell infiltration using the TARGET RNAseq data. We first used ESTIMATE to predict percent tumor purity and found predicted purity was generally high across all samples (interquartile range = 0.72–0.85) and anticorrelated with predicted overall immune infiltration of 22 immune cell types by CIBERSORTx (ρ = −0.510, *p* = 1.4 × 10^−6^). CIBERSORTx predicted M2 followed by M0 macrophages to be the most abundant immune cell types across tumors based on median predicted values. Overall, variation in immune cell composition was low, as CIBERSORTx predicted immune infiltrate was not significantly different between tumors in the main cluster groups defined by the Global, RFS, or CR profiles (min. *p* = 0.534, Supplementary Table 5, Supplementary Fig. 7). However, predicted immune infiltrate was significantly greater in the EPIMMUNE derived cluster group with lower tumor purity (*p* = 0.035), potentially due to increased *CD4* memory resting T cells (*p* = 0.049) or resting mast cells (*p* = 0.002). Taken together, Global and outcome profile methylation appear to offer information independent of immune cell infiltration, but DNA methylation could still be useful for the precision application of immunotherapy.

### Biologic and functional annotation of the methylation profiles

**Fig. 7:**
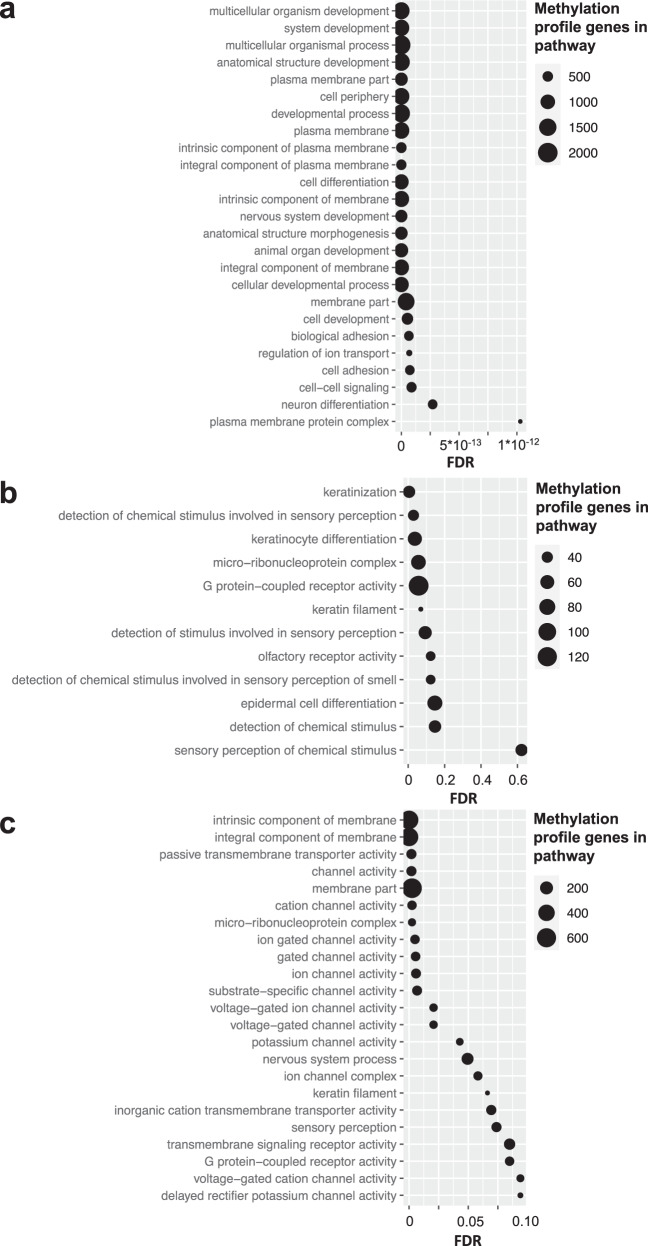
*MissMethyl gometh* pathway enrichment analysis of the methylation profiles. Terms with FDR < 0.1 were considered enriched. **a** GO terms enriched in the Global profile. The top 25 most significantly enriched terms are shown. **b** GO terms uniquely enriched in the sites differentially methylated between the Global profile cluster groups compared to the full profile (**a**). **c** GO terms enriched in the sites associated with chemoresponse.

In order to gain insight into the methylation profile content we used the *missMethyl gometh* gene-set analysis function^57,58^. We first tested if Gene Ontology (GO) and Kyoto Encyclopedia of Genes and Genomes (KEGG) terms were enriched in the Global profile. The most enriched terms were related to developmental pathways, e.g. “multicellular organism development,” “system development,” and “anatomical structure development” (Fig. 7). Bone Morphogenic Protein (BMP) related terms such as “Response to BMP,” “cellular response to BMP stimulus” and “Cytokine-cytokine receptor interaction” were also identified. Other enriched pathways include “plasma membrane parts,” and “Cell adhesion molecules” notable because these play a role in therapy resistance and metastasis^59,60^.

While no terms were enriched in the RFS and CR signature when correcting for multiple testing (FDR < 0.1), this was likely due to the refined nature of the signatures with too few CpG sites in the analysis (Supplementary Data 4). In a more inclusive analysis of all associated CpG sites, we found that the CR profile was enriched with membrane components and channels, similar to the Global profile. While the number of RFS associated sites was still small, analysis using a relaxed statistical cutoff (uncorrected *p* < 0.05) identified a list of terms highly non-overlapping with CR profile (hypergeometric *p* < 1 × 10^−9^), including pathways commonly dysregulated in cancer, suggesting the profiles capture unique biological features. Finally, we tested the sites differentially methylated between the Global profile cluster groups (Fig. 1b). The most enriched terms were again largely related to the plasma membrane and membrane receptor/channel signal transduction. Twelve of the 61 GO terms were non-overlapping with those enriched in the Global profile, and among them, four are related to epidermal cell differentiation. Notably, sarcomas have been postulated to exist in a metastable state, able to switch between mesenchymal or epithelial-like states, and OSAs sometimes even stain positive for cytokeratin, factors related to tumor behavior^61–63^. “Micro-ribonucleoprotein complex” (the RISC complex) was also among the 12 unique terms, notable because we have previously reported miRNA expression defines prognostic groups in OSA, and miRNAs are known developmental regulators related to mesenchymal differentiation^27,64^.

### The RFS methylation markers may regulate a transcriptional program also prognostic of survival

We studied the potential downstream consequences of methylation using RNA sequencing data available for the same TARGET samples. Of the methylation markers, 71.7 and 54.0% of the RFS and CR profiles, respectively, were annotated to specific genes, and 39.8% of the profile sites correlated with their annotated genes (Spearman correlation, FDR < 0.1, Supplementary Data 5). Additionally, correlations were stronger where the CpG site was annotated to the gene (expected cis-level interaction) compared to where the CpG site was not annotated to the gene (average |ρ_expected_ | = 0.221, average |ρ_other_ | = 0.138, *p* = 1.1 × 10^−60^, Supplementary Fig. 8). The significant correlations for the RFS and CR profiles were 60.7 and 8.22% negative, respectively. The prevalence of positive correlations could be partly explained by the large number of gene-body methylation sites in our profiles, containing 75.2% and 82.2% gene-body sites in the RFS and CR profiles, respectively. Contrary to promoter methylation, gene-body methylation can be a mark of active transcription^65^. Additionally, the small fraction of 3′ UTR site in the signature were all positively correlated with transcription, an observation reported in multiple adult cancer types^66^. We tested if the genes correlating with methylation were differentially expressed between the methylation-based cluster groups. More genes were significantly correlated with methylation and differentially expressed (FDR < 0.1) between the methylation-based cluster groups for the RFS profile than the CR profile (53 of 219 and 7 of 142 genes, respectively). These results suggest that many of the RFS methylation markers likely regulate transcription, but a much smaller subset of the CR profile is directly connected to downstream gene expression, thus the CR profile potentially represents a wider “cell state” rather than acting through a few key specific mechanisms. We also tested for differential expression between the Global profile cluster groups (Supplementary Data 5). Of the 149 genes differentially expressed, 18 were cancer-testis antigens encoded on the X chromosome (including *MAGE*, *CSAG*, *PAGE*, *SSX*, and *XAGE* gene family members), all upregulated in the good prognosis group in Fig. 1b.

**Fig. 8:**
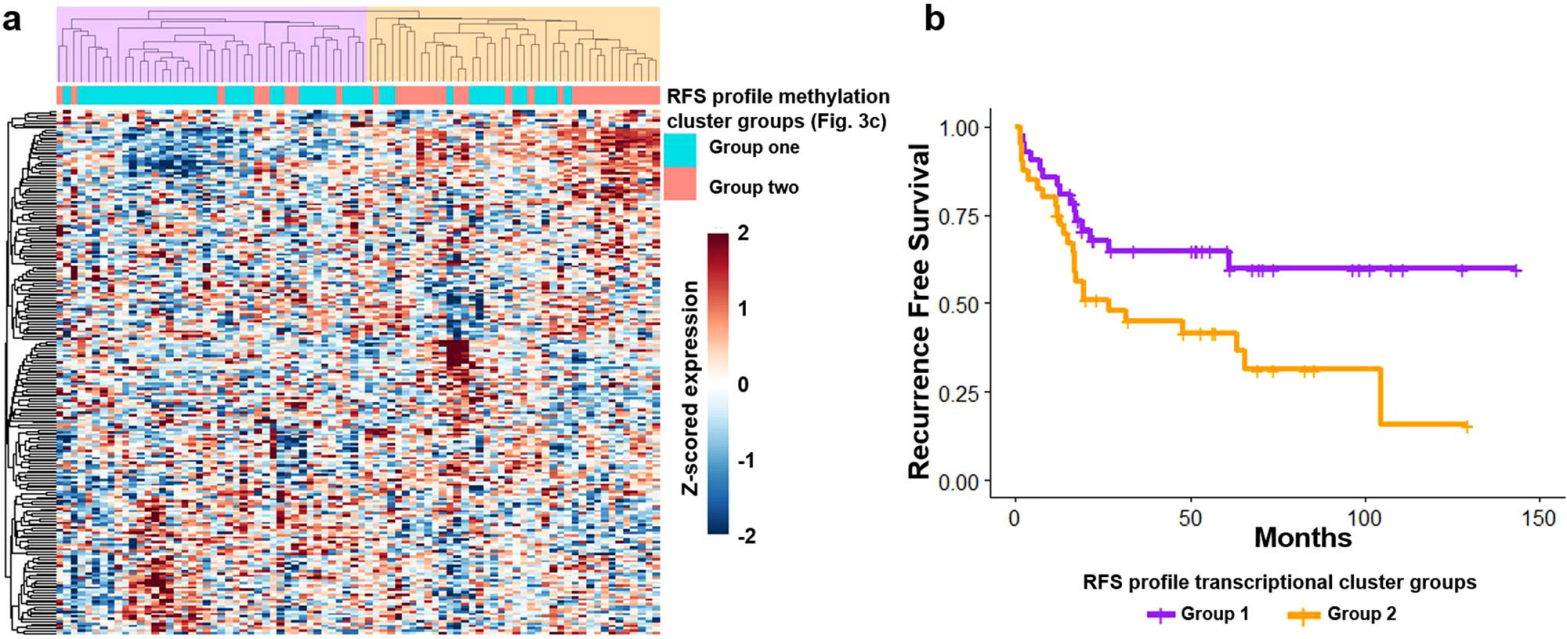
Transcription of genes annotated to the RFS profile. **a** Hierarchical clustering using transcriptional of genes annotated to the RFS profile (2-group R-index = 0.78). Classification is similar to the respective RFS methylation cluster groups (Fig. 3c, Cramer’s *V* = 0.443, *p* = 1 × 10^−4^). **b** RFS analysis using the two main transcription-based cluster groups (Log Rank *p* value = 0.024, median RFS: NYR (Group 1, purple) vs. 26.7 mo. (Group 2, orange)).

We also examined whether transcription at the methylation profile loci was associated with clinical outcomes. We found that the sets of genes annotated to the methylation profiles were significantly associated with the same clinical outcome predicted by the respective methylation profile using geneset enrichment analysis (GSA, LS permutation *p* < 0.05, Supplementary Data 6)^67^. Of note, a large fraction of the CR profile sites were not annotated to a known gene and were located in so-called gene deserts which are enriched in the Open Sea genomic region^68^, potentially explaining why transcription from CR profile loci was not as strongly associated with clinical outcome. As above, this suggests that a broad cell state may be influencing response to chemotherapy. We also found that transcription of genes annotated to the RFS profile group samples similar to the profile’s methylation patterns by hierarchical clustering (Fig. 8a, Cramer’s *V* = 0.443, *p* = 1 × 10^−4^). Finally, a significant survival difference was observed between the two main RFS profile transcriptionally defined hierarchical clustering subgroups (Fig. 8b), strengthening the connection between the RFS methylation markers and downstream transcriptional regulation. We did not find this concordance for the CR signature (Fisher’s *p* = 0.31) further underscoring that chemotherapy response is correlated with very broad methylation patterns rather than regulation of a few specific genes.

We then tested GO and KEGG terms also using GSA analysis and found transcription of many terms associated with the RFS (Supplementary Data 6). We found many fewer transcriptional pathway associations with the CR than RFS profile in the GSA analysis, further evidence that methylation may be predicting chemotherapy response independent of known regulatory pathways.

### In vitro correlates of the clinical methylation profiles

**Fig. 9:**
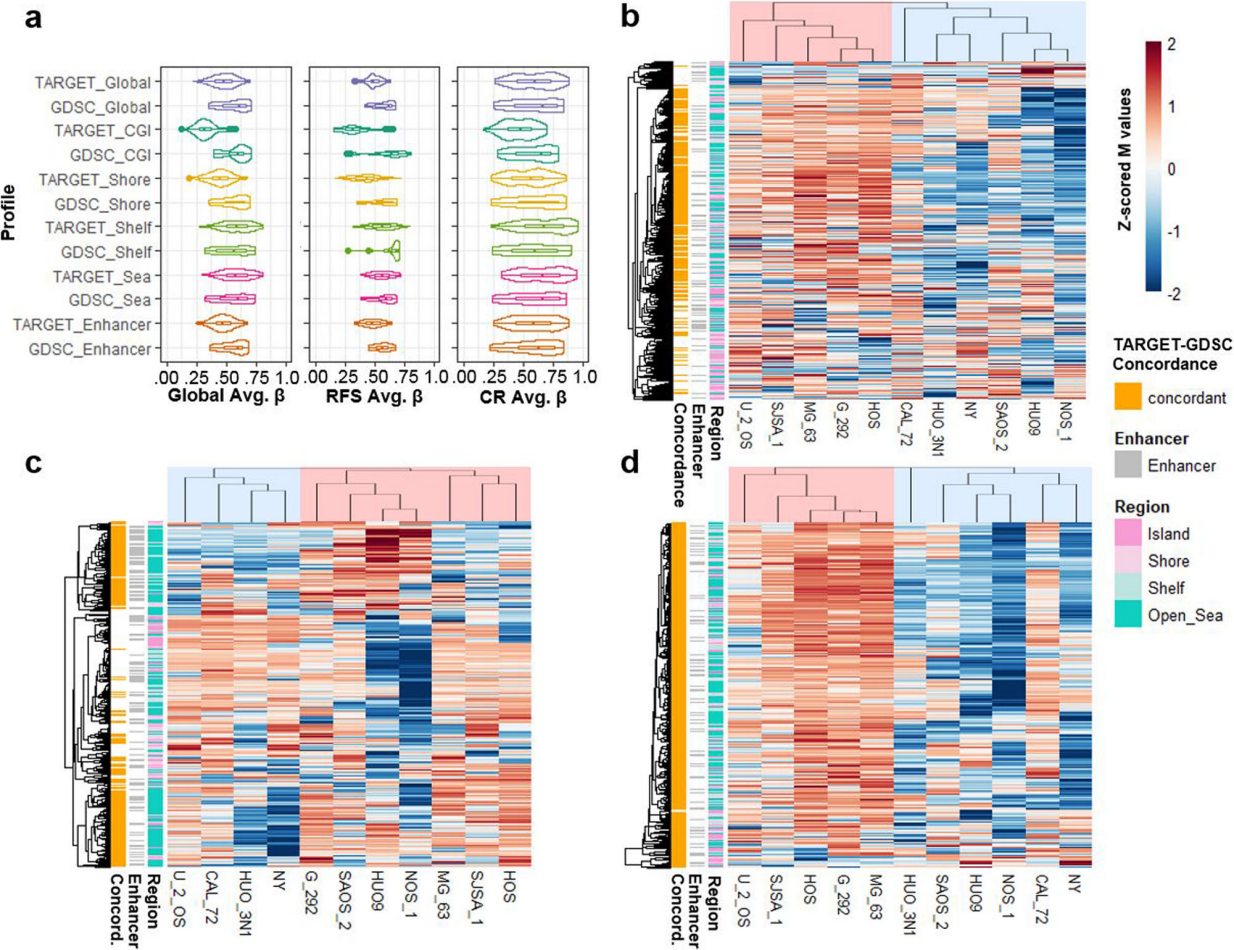
Methylation profiles in OSA cell lines. **a** Violin plots of the average β value distributions of the Global, RFS, and CR profiles and region-specific subsets in the GDSC cell lines and TARGET (for comparison) datasets. Boxplots depict the median, interquartile range (IQR), and 1.5 * IQR. **b**–**d** Semi-supervised hierarchical clustering of the 11 OSA cell lines in the GDSC dataset using the Global (**b**), RFS (**c**), and CR (**d**) profiles. CpG sites displaying concordant hypo/hyper-methylation patterns in the cell line GDSC dataset relative to the TARGET clinical dataset are annotated in the first row annotation track of each heatmap. Detailed concordance results are presented in Supplementary Table 6.

We used public in vitro multi-omic and pharmacologic dataset (Genomics of Drug Sensitivity in Cancer (GDSC))^69–71^ including 11 OSA cell lines (all from cases <20 years old) to assess in vitro correlates of the clinically derived methylation patterns as a prelude for future experimentation and therapeutic discovery. We compared the average β values of the Global and outcome profiles of the GDSC and TARGET datasets (Fig. 9a) and found that the methylation levels of the cell lines were similar to those of the clinical samples, albeit with a trend for CGI hypermethylation in the cell lines, as observed in other cancer types^72,73^. We also assessed the similarity of in vitro methylation patterns using semi-supervised hierarchical clustering (Fig. 9b–d) and compared differential methylation between the two primary cluster groups using the same methodology used to compare the clinical datasets. The Global and outcome profiles’ methylation patterns were similar between the in vitro and clinical datasets (Fig. 9 and Supplementary Table 6). The RFS profile displayed a balance of hyper and hypomethylation, and the Global and CR profiles were largely hyper or hypomethylated, similar to what was seen in the clinical datasets (Figs. 1, 3–5).

Cell line aggressiveness metrics, including proliferation, invasion, migration, colony-forming ability, and tumorigenicity, were previously reported for seven of these OSA cell lines^74^. We tested the methylation profiles in relation to these cell line metrics. Given the very small number of cell lines, proper correction for multiple testing was challenging. While we found that a relatively small fraction of individual CpG sites in our RFS and CR profiles correlated with in vitro aggressiveness (Spearman, *p* < 0.05), testing 1000 randomly selected CpG site sets of equal size to the outcome profiles indicated that RFS profile is enriched (permutation *p* < 0.1) for sites correlated with migration, and proliferation, and the CR signature is enriched for sites associated with colony formation (Supplementary Table 7).

The same GDSC datasets also provided in vitro drug response testing for standard chemotherapeutic agents used in OSA (MAP)). The three components of MAP were not tested in combination in vitro, (as they are always used clinically). Thus, we created a surrogate MAP score variable by scaling each cell line’s response to each drug to a value between 0 and 1, where 0 and 1 were assigned to the cell lines least and most resistant to the drug, respectively, and then averaged the values to generate the scores. We found a small number of individual CpG sites associated with the surrogate MAP score in vitro (Supplementary Table 8). Permutation testing indicated the RFS profile but not the CR profile was enriched for sites correlated with doxorubicin and methotrexate and the MAP score. Given the known association between chemotherapy response and patient survival, it was not surprising the RFS profile was associated with in vitro drug response. The lack of clear associations with the CR profile may suggest the profile predicts an interaction between all three drugs, (which were tested in isolation in the GDSC dataset), and/or that the RFS profile may be a better candidate for future drug development efforts.

### Large-scale integrative pharmacogenomic analysis identifies drugs with potential activity in OSA

We tested if the RFS profile can be used to predict response to drugs and for drug repurposing. There is currently no analytical pipeline optimized for methylomic patterns for pharmacogenomic analysis. Therefore, we used transcription from the profile loci as a surrogate. We did not use the CR profile as there was no clear transcriptional correlate for the broad profiles of chemoresponse and we did not see a robust signal for in vitro association with standard therapies. We used the comprehensive pharmacogenomic analytical platform PharmacoGx, via its PharmacoDB interface^75,76^, which we have previously used for drug screening^27,77^.

Among the genes annotated to the RFS profile, we selected the 47 most highly correlated with their methylation markers (*p* < 0.001) reasoning expression of these genes would be the best surrogate for the methylation profile. We tested the 47 genes for drug response association in a pan-cancer analysis using the 1691 cancer cell lines and 759 compounds included in the database. Initial drug selection based on a regression analysis identified 61 drugs. Then, we focused our analysis on the subset of 11 OSA cell lines to increase specificity and eliminated drugs with the least variant IC50. We further selected drugs with largely concordant direction of the drug response/gene expression association between the pan-cancer and OSA-specific analysis. For increased potential clinical impact, we considered drugs more potent than cisplatin in at least two of 11 OSA cell lines. The resulting list of 18 drugs is presented in Table 1. We then used the in vitro GDSC methylomic dataset to search for more direct evidence of relationships between drug response and methylation, and we found that for six of these drugs the RFS profile had more significant correlations with response than 90% of 1000 random CpG site sets of equivalent size (permutation *p* < 0.1). A variety of pharmacologic mechanisms characterize the drugs included in Table 1 and the drug/combination with the most significantly correlated sites includes a methyltransferase inhibitor, UNC0638, which has epigenetic modifying effects.

**Table 1:** Pharmaceuticals for which PharmacoDB analysis reveals a predictive drug response association with the transcription from the RFS profile loci.

Drug	Median IC50 (μM)	Median regression coefficient	Mechanism of action
GMX-1778	0.021	0.190	NAMPTi
tanespimycin + **gemcitabine**	1.189	0.175	HSP90i + nucleoside analog
**CUDC-101**	1.634	0.196	HDACi + EDGFR/HER2i
alistertib + navitoclax	1.692	0.194	AURKAi/BCLi
mirdametinib	2.863	0.248	MEKi
**vorinostat** + navitoclax	3.116	0.162	HDACi/BCLi
ceranib-2	4.434	0.188	ceramidase i
BRD-K34222889	4.464	0.195	GSTP1i
navitoclax + piperlongumine	4.976	0.178	BCLi/GSTP1i
necrosulfonamide	6.260	0.194	MLKLi
Genetech Cpd 10	7.190	0.190	AURKA/Bi
**UNC0638** + navitoclax	9.552	0.230	G9ai/GLPi + BCLi
navitoclax + sorafenib	10.182	0.186	BCLi + c-RASi
alisertib	10.196	0.184	AURKAi
BX-912	11.335	0.182	PDK1i
CL-1040	12.805	0.188	MEKi
carboplatin + etoposide	19.099	0.207	DNA crosslinking + TOPIIi
tretinoin + navitoclax	27.382	0.188	RAR/TERTi + BCLi

Median IC50 values are obtained across all OSA cell lines in the GDSC dataset though PharmacoDB. The median regression coefficient is calculated from the significant gene–drug interactions used to identify the drugs. Bolded drugs act through epigenetic mechanisms. Underlined drugs have more significant response correlations (*p* < 0.05) with the RFS methylation profile than at least 90% of 1000 randomly generated CpG site sets in OSA cell lines.

Given the recent interest in cabozantinib and pazopanib in OSA, we also performed a targeted test for association between the RFS profile and response to these drugs^78–83^. In a prespecified hypothesis-based analysis, we found that transcription of one and two of the RFS profile genes were associated with cabozantinib and pazopanib response, respectfully. When we evaluated the response associations, specifically in the OSA cell lines, we found that the direction of the gene-response association for cabozantinib, but not those for pazopanib, was concordant with the pan-cancer analysis. Finally, in the GDSC methylomic data, we found more RFS profile sites significantly correlated with response to cabozantinib than all 1000 randomly generated CpG sets.

## Discussion

Despite ongoing efforts, there has been no meaningful advancement in OSA therapies or biomarkers for the last three decades. Conventional genetic studies have not uncovered actionable drug targets, and the only accepted prognostic factor is pathologically assessed tumor necrosis following standard neoadjuvant therapy. This did not lead to improvement in survival when used to stratify patients in a recent large randomized international trial (EURAMOS)^6,22,23^ and recent consensus has emerged that there is a pressing need for robust biomarkers that better align with biologic and clinical heterogeneity in this tumor^84,85^. The state of DNA methylation is often disrupted in cancer^86^. Furthermore, DNA methylation carries prognostic information and defines disease subtypes with different treatment response in other cancers^44,87,88^. In OSA, prior reports have described single gene or locus methylation affecting progression or treatment response^30,31^. A pilot clinical study of 15 patients suggested that the genome-wide DNA methylation state of pre-treatment OSA tumors is potentially associated with outcome though the small sample number did not allow full statistical assessment of these patterns^36^.

We analyzed the multi-omic NCI TARGET OSA dataset (recently made fully publicly available), representing the largest (*N* = 83 with survival annotations) methylation profiling study thus far in this relatively rare, mainly pediatric disease. Genome-wide analysis revealed that primary tumor methylation patterns were strongly associated with patient outcomes. Most striking was the large genome-wide difference in methylation state between tumors which did and did not respond to standard therapy, with the relatively hypomethylated tumors responding better to chemotherapy than the hypermethylated tumors. This was found by both unsupervised clustering, and a supervised approach whereby the methylation level of genome-wide CpG sites was simply averaged for each sample, suggesting that the clinically relevant DNA methylation patterns are present across a large fraction of the genome, and not limited to a few genomic loci. Notably, broad genomic hypomethylation is known to cause genomic instability and increase tumor immunogenicity. This is especially interesting in light of recent immunogenomic findings suggesting that OSA tumors with optimal chemoresponse have less stable genomes and higher activation of immune response than those with suboptimal response^89^. In light of this, we nevertheless found that the methylation profiles were not driven by differences in immune cell infiltration.

Methylation patterns were also found to predict long-term survival. Unsupervised clustering of sample groups revealed that the same genome-wide methylation patterns were also prognostic of RFS and OS when stratified for the presence of metastasis at the time of diagnosis. When we focused on specific regions of the genome based on CpG density, we found that methylation of CGIs was most strongly prognostic of long-term outcome, although distinct from the well-known CRC and glioma CIMPs. We found that CGI methylation patterns within a given risk group were heterogenous, unlike the CpG sparse genomic regions (Open Sea) which were more uniformly hyper or hypomethylated in a given methylation subgroup. It is, thus, conceivable that CGI methylation contributes to locus-specific regulation mediating gene transcriptional programs, whereas Open Sea regions represent a biologically meaningful broader genomic state.

In addition, a supervised RFS prognostic profile was discovered with strong FDR corrected statistical significance and validated in an (albeit small) independent dataset, although a fully defined model based on this profile could not be tested in this independent dataset given the very different methylation assay used. Analysis of this RFS profile revealed a subpopulation of CpG sites where the CGIs were primarily hypomethylated, and Open Sea sites were primarily hypermethylated, in a poor prognosis subgroup. Further support for the notion that different regions offer non-overlapping prognostic information was obtained through multivariate analysis of the RFS profile while controlling for response to chemotherapy. CGI sites were depleted in the RFS profile but enriched when only considering the subset of sites predictive of outcome independent of chemoresponse, and even suggested an inverse methylation-outcome relationship (with hypermethylation associating with better outcome) compared to the genome-wide pattern. This contrasted with the chemoresponse (CR) profile, which showed nearly ubiquitous hypermethylation in the poor response group, except for a very small subset of CGI and Shore sites. The cancer genome is generally known to gain methylation at CGIs and lose methylation in Open Sea regions^51^, thus it is interesting to observe that our signatures predict that tumors with methylation patterns more reminiscent of normal tissue may be more aggressive and less responsive to standard chemotherapy.

While have previously reported that miRNA patterns are prognostic of survival, we have found limited evidence to date for a robust and reproducible miRNA signature associated with chemotherapy response. Our results suggest that the methylation profiles are offering independent, and more powerful chemoresponse prediction than miRNA transcription. In this respect, multivariate analysis also demonstrated that the prognostic power of the RFS profile was independent of our previously published miRNA survival signature. Thus, these different omics patterns can be potentially used in a complementary manner to identify clinically relevant tumor subtypes in OSA.

Analysis of three additional independent publicly available but relatively small clinical datasets allowed us to demonstrate the reproducibility of the methylation profiles. Independent validation of the clinical predictive and prognostic value of the profiles was performed in the AECM HELP-tagging dataset, despite the small number of samples and the completely different profiling platform that was used in that dataset. The incorporation of two additional OSA 450k array datasets demonstrated that the molecular information carried in each dataset could be used to group samples with similar methylation patterns as observed in the large TARGET cohort, suggesting the methylation profiles may indeed define reproducible molecular subtypes. Furthermore, our results extend upon those from the previous study of outcome relevance of DNA methylation, which profiled 15 samples using a HELP-tagging assay, and found increased genome-wide DNA methylation in primary tumors of patients that eventually relapsed compared to those that remained in remission (though the relatively small sample size had limited definitive statistical conclusions)^36^. This is now proven by the finding from our adequately powered genome-wide clustering analysis where the hypermethylated subgroup displayed worse RFS. In addition, we show a striking prediction of chemoresponse by DNA methylation.

When analyzing the relationship between the profile CpG sites and known genes, we found many sites were intra- as well as intergenic. Gene expression analysis of the same samples profiled in the TARGET methylation cohort revealed a possible connection between the methylation profiles and transcription from their genomic loci. This connection was quite robust for the RFS profile, where methylation and downstream transcription profiles were found to carry similar prognostic information, suggesting methylation may be activating or repressing key genes. The connection between the CR profile and transcription was weaker, suggesting that the larger epigenomic cellular state may be a better marker for response to standard therapy than a methylation effect on a few specific molecular pathways.

We used transcription from the RFS profile loci to perform pharmacogenomic drug discovery analysis using a integrative bioinformatic pipeline^75,76^ and identified a set of drugs for which methylation patterns might serve as response biomarkers. These drugs were almost entirely non-overlapping with a set of candidate drugs from a similar analysis we previously reported which used the gene targets of prognostic miRNA profiles, suggesting again that methylation and miRNA patterns offer non-redundant clinical applicability^27^. Several of these drugs act through epigenomic mechanisms, especially modulation of post-translational histone modifications. Histone modifications influence and can be influenced by the DNA methylation state of the underlying locus and provide modifiable regulation of gene expression, an active area of research in other solid and hematological malignancies^90^. Notably, the *HDAC4* gene (the protein product of which deacetylates core histones) and the gene of its binding partner *MEF2C*^91^, were found in the RFS profile, which also included five replication-dependent core histones (H2A, H2B, H3, and H4) from the H1 histone cluster on chromosome 6^92^. Three of the histone genes identified in the signature (*HIST1*, *H2BK*, *HIST1H3J*, and *HIST1H4I*) have been shown to have dysregulated transcription in other tumors^93^. The *HIST1H4I* gene has been shown to be differentially methylated in parathyroid tumors compared to normal tissue^94^. Mutations of H3 histones are highly prevalent in other bone tumors such as giant cell tumors of bone, chondroblastomas, and chondrosarcomas and are also found in pediatric brain tumors and were recently also identified in a small subset of OSAs^95–100^. These H3 mutant tumors displayed different methylation patterns compared to H3 wildtype OSAs, and one of the two most differentially methylated sites was in the histone micro-cluster included in the RFS signature^97^. Our finding that imprinted genes and those monoallelically expressed are among the most differentially methylated between the Global cluster groups suggests that these regions may play a critical role in not only tumorigenesis, as has already been reported in OSA and other cancers, but also tumor behavior and patient outcome. Additionally, the large methylation differences at the chromosome 6 HLA locus potentially indicates immunological mechanisms may play a role in patient survival, even if immune infiltrate is relatively invariant across tumors.

Demethylating drugs have shown pre-clinical efficacy in OSA^30,31^. Furthermore, standard chemotherapy includes cisplatin and doxorubicin, both of which induce direct DNA damage. Hypermethylation may protect against these insults or even physically disrupt their mechanisms by limiting chromatin accessibility or stabilizing the genome. Moreover, epigenetic targeting has been shown to potentiate chemotherapy effect, and a recent study reported that a DNA methyltransferase inhibitor synergized with both doxorubicin and cisplatin in OSA cell lines^101,102^. Furthermore, HDAC inhibitors synergize with doxorubicin in short-term cultures derived from orthotopic patient-derived xenograft models^103^. Finally, our finding that the EPIMMUNE signature, recently reported predicting immunotherapy response in lung cancer, is detectable in a subset of osteosarcoma samples merits further dedicated investigation. In conclusion, our study implies that epigenetic therapies hold promise for synergistic combination with standard chemotherapy and be especially useful in the subset of hypermethylated tumors expected to respond poorly to chemotherapy

Limitations of our study include the limited sample size and different methylation assay used for the validation dataset with available patient survival data, and the lack of survival data in the other two independent validation datasets. Additionally, we could not evaluate a possible association with histologic subtypes given the lack of such information in any of the public datasets that were available. Further validation, optimization and refinement, and final assay selection will be needed prior to the clinical application of the methylation profiles described in this report. These questions will require larger study cohorts and we anticipate our report will facilitate the planning of such collaborative and costly new studies.

Standard clinicopathologic variables alone have thus far not been adequate as markers for improved or properly tailored therapies for OSA, highlighted by the failure of the recent EURAMOS trial^6,23^, which used pathologically assessed tumor necrosis to stratify patients for alternate or intensified therapy. DNA methylation profiles herein, may ultimately offer a powerful and biologically informed method to complement clinical prognostic factors for therapeutic stratification. Additionally, recent work has shown that tumor DNA methylation patterns are preserved in formalin fixed paraffin embedded (FFPE) tissue facilitating the study and application of methylation markers in rare tumors like OSA^104,105^, as well as in the blood stream^106,107^, such that minimally invasive methylation assays assisting clinical management of OSA may ultimately become feasible.

## Methods

### Public data acquisition

We used the following publicly available published or reported datasets (TARGET, AECM, JNCCRI, NY, and GDSC)^30,35,36,108^. All data analyzed was publicly available and experiments were approved by the presiding Institutional Review Board where each dataset was generated and informed consent was obtained where required. Ethics statements can be found in the source publications. All experiments were performed in accordance with relevant guidelines and regulations. Sample selection criteria are available in the source publications and at the TARGET initiative website (https://ocg.cancer.gov/programs/target/projects/OSA). The TARGET dataset was downloaded from the TARGET data matrix (https://ocg.cancer.gov/programs/target/data-matrix). The AECM (GSE59200), JNCCRI (GSE125645) and NY dataset (GSE97529) were downloaded from the Gene Expression Omnibus.

### Illumina Infinium HumanMethylation450 BeadChip data processing

The TARGET, JNCCRI, NY, and GDSC datasets assessed genome-wide methylation with the Illumina Infinium HumanMethylation450 BeadChip platform. Array CpG annotations were obtained from the HumanMethylation450 v1.2 manifest file available at https://support.illumina.com/downloads/infinium_humanmethylation450_product_files.html. We downloaded preprocessed β values (methylated signal/(methylated signal + unmethylated signal)) for each clinical dataset. We also used *minfi*^40^ with functional normalization^109^ to process the raw signal intensity (idat) files for the TARGET dataset to ensure the results obtained were not dependent on the specific preprocessing method used (Supplementary Note 1). Technical details of β value generation are available in the source publications and at the TARGET initiative website (https://ocg.cancer.gov/programs/target/target-methods). Standard pre-processing including out-of-band signal intensity correction, Lumi dye bias color correction, and beta mixture quantile dilation probe bias normalization was already applied to the preprocessed TARGET dataset. Analysis of the TARGET dataset was performed using the 83 samples for which survival information was available. Data generated by probes containing frequent SNPs and those targeting the sex chromosomes were omitted from the preprocessed TARGET dataset^110^, and we did not consider them in our analysis of the other datasets. Data was additionally processed by converting β values to M values. M values were used for all analyses except where explicitly stated. M values have been shown to better meet assumptions for various parametric statistical tests than β values^111^. A filter selecting the 5% most variant CpG sites across the TARGET dataset was used for discovery analysis to reduce statistical noise. The other datasets were analyzed using sites identified in the TARGET dataset. We downloaded idat files for OSA cell lines available in the GDSC dataset. Detailed methodology is available in the source publication. β and M values were then generated via the *minfi* R package using Functional Normalization (an Illumina 450k array adapted quantile normalization) with default settings.

### Gene transcriptional analysis

RNA sequencing data was available for the TARGET dataset. The detailed methodology can be found on the TARGET initiative website (above). In summary, RNA was poly-A purified, gene libraries were prepared and multiplexed following standard Illumina protocol, and sequenced using the Illumina HiSeq 2000 platform. Transcript abundance was quantified with kallisto^112^. Count-level data was downloaded then normalized and log base 2 transformed via the DESeq2 R package^113^.

Affymetrix Human Genome U219 transcription array data was available for the GDSC cell line dataset. Robust Multi-array Average (RMA) normalized transcription array data for the GDSC cell line dataset was downloaded from the project website (above)^114^. The detailed methodology can be found in the source publication.

The TARGET dataset assayed miRNA abundance using the ABI TaqMan Megaplex human miRNA qRT-PCR platform. Technical details are available on the TARGET initiative website (above). We transformed and normalized raw miRNA qRT-PCR data using standard 2^−ΔCt^ transformation as previously described^115^.

### Unsupervised and Supervised Survival analysis and prediction

Hierarchical clustering analysis was performed with the centered correlation and average linkage method for 450k array datasets and Euclidean distance and complete linkage for the HELP-tagging dataset. The resulting subgroups were then analyzed for survival differences^116^. Cluster reproducibility was assessed with the R-index^117^. Heatmaps display mean centered and standard deviation scaled methylation patterns for M value and transcriptional data, or raw methylation values where noted. Methylation-based supervised analysis was performed using an average β approach whereby the average β value for each sample was calculated, and then samples were classified into two groups based on the median average β value. Survival differences between cluster or average β defined groups were tested with Kaplan-Meier analysis and the log-rank test for significance. The sample cluster groups in the independent datasets were determined to be either more similar to cluster group 1 or group 2 from the TARGET dataset by maximizing the fraction of sites with a concordant direction of fold change which were significantly differentially methylated in both datasets. The cluster groups in the independent datasets were then compared using the group assignments based on comparison with the TARGET dataset. MiRNA based supervised analysis was performed using the signed average approach as previously described^25,27^. Multivariate analysis for confounding prognostic factors was performed using a Cox regression model, with the methylation signature and relevant factors entered as independent co-variates.

### Statistics and reproducibility

Two groups continuous variable differential analysis was performed by a t-test with p values adjusted to control the false discovery rate using the Benjamini-Hochberg step-up procedure for multiple testing^37^. CpG sites specific differential methylation testing used M values. Region-based differential methylation analysis was performed using *minfi*^40^ and *bumphunter*^41^ with a beta value difference cutoff of 0.1, a maximum gap between sites to define clusters of 500 bp, loess smoothing by cluster, and 1000 permutations. Gene set analysis for association with outcome was performed with the functional class scoring method, applying the LS test with permutation-based *p* values, and only considering terms with at least five genes in the gene set being tested^67^. Associations between two categorical variables were evaluated with two-tailed chi square/Fisher’s exact test and Odds ratio. Cramer’s *V* test was used to assess the strength of the classification concordance between different profiles. Spearman’s rho statistic was used to evaluate continuous variable correlations, except for gene selection for the drug discovery analysis, which used Pearson correlations. The hypergeometric test was used to test enrichment or depletion. To assess specific associations between the methylation profiles and cell line aggressiveness and response to therapy in vitro we performed a simulation test using 1,000 sets of 374 CpG sites (the number of sites in the outcome profiles) randomly selected from the 5% most variant sites in the TARGET dataset. We then correlated methylation of the random CpG sites with cell line aggressiveness metrics and response to therapy and quantified the number of significant interactions (*p* < 0.05). Sample size information is available in the text and Supplementary Data 1. No technical replicates were analyzed. Individual tumor samples or cell lines sharing a common phenotype or characteristic were analyzed as biological replicates.

### CpG island methylator phenotype score calculation

Panels of genes used to define CIMP in CRC and glioma were obtained from prior reports^44–46^. The CIMP panels reported by Toyota et al. (“Toyota panel”), consisting of the *CDKN2A*, *MINT1*/*APBA1*, *MINT2*/*APBA2*, *MINT31*/*CACNA1G*, and *MLH1* genes, and Weisenberger et al., (“Weisenberger panel”), consisting of the *CACNA1G*, *IGF2*, *NEUROG1*, *RUNX3*, and *SOCS1* genes were used to test for CRC CIMP like methylation. The eight gene panel reported by Noushmehr et al. (“Noushmehr panel”), comprised of the genes *ANKRD43*/*SOWAHA*, *HFE*, *MAL*, *LGALS3*, *FAS-1*/*FAS*, *FAS-2*, *RHO-F*, and *DOCK5*, was used to test for glioma CIMP like methylation. *DOCK5* was reported by Noushmehr et al. to display opposite methylation patterns of the other genes in the glioma panel (hypomethylated in CIMP + tumors), so we used 1- β values for *DOCK5* sites when calculating the CIMP score.

The CIMP panels were measured in the TARGET dataset by calculating the average β of CGI sites in the panel genes for each patient (CIMP scores). 73, 140, and 44 sites were used for the Toyota, Weisenberger, and Noushmehr panels, respectively. Separate scores were also calculated for the panel using only promoter-associated sites.

### Immunogenomics

CIBERSORTx^55,56^, was used to predict the abundance of 22 types of potentially infiltrating immune cells in primary OS tumors. Count-level RNA-seq data filtered for Ensembl genes with HGNC symbols (*n* = 24,865) from tumors with methylation profiling in the TARGET dataset (*n* = 82) was used as the mixture file for the CIBERSORTx Cell Fractions Analysis module. LM22 was used as the signature matrix, and the LM22 Source gene expression profile was used as the source gene expression profile file to predict the abundance of 22 immune cell types. Quantile normalization was disabled, and the analysis was run in absolute mode with 100 permutations. Tumor samples for which CIBERSORTx predicted immune cell infiltration above what would be expected due to chance (all 82 analyzed samples, permutation *p* < 0.05) were used for statistical analysis.

ESTIMATE^54^ was used to estimate sample tumor purity for each of the 82 samples in the TARGET dataset with both methylation and RNAseq data using the same 24,865 genes used for CIBERSORTx analysis.

### Methylation profile pathway enrichment analysis

Enrichment of Gene Ontology (GO) and KEGG terms in the outcome profiles was tested using the *missMethyl* R package *gometh* function^57,58^. GO and KEGG terms with an FDR < 0.1 were considered enriched.

### Integrative pharmacogenomic analysis

We used transcription of the genes annotated to and most highly correlated with the RFS profile (CpG methylation-gene transcription *p* < 0.001, *N* = 47) for drug discovery using the PharmacoDB interface, which performs data analysis via the PharmacoGx R package^75,76^. The 47 genes were entered in the PharmacoDB pipeline analyzing gene–drug predictive interactions from seven large-scale datasets including a total of 650,894 individual drug sensitivity experiments, 1691 cell lines, and 19,933 gene markers via a multivariate regression model adjusting for tissue source and experimental batch. We chose drug candidates at a stringent 0.001 regression p-value for predictive association with gene markers, and a regression coefficient > |0.15 | (coefficient > |0.1| for hypothesis driven analysis) for effect size in a pan-cancer analysis, which used all cell lines in the database for increased sensitivity compared to the few OSA cell lines. To increase the specificity of the resulting drug list, only drugs passing the p-value and effect size filters for at least three of the tested genes were retained for further analysis. The in vitro experimental data of the filtered drug list was then evaluated specifically in the 11 OSA cell lines contained in the GDSC dataset^69^ through the PharmacoDB Batch Query to obtain sensitivity measures (IC50 dose response metric) for response to candidate drugs. We assessed gene–drug correlations in the small subset of OSA cell lines and required at least two-thirds of the interactions to have the same correlation sign as those observed in the pan-cancer (1691 cell line) analysis. We finally selected drugs with the most variant IC50 response (top two tertiles) across OSA cell lines, and those more potent than cisplatin in at least two OSA cell lines.

### Reporting summary

Further information on research design is available in the Nature Research Reporting Summary linked to this article.

## Supplementary information

Supplemental Material

Description of Additional Supplementary Files

Supplementary Data 1

Supplementary Data 2

Supplementary Data 3

Supplementary Data 4

Supplementary Data 5

Supplementary Data 6

Reporting Summary

## Data Availability

The datasets analyzed in this study are available at the TARGET data matrix (https://ocg.cancer.gov/programs/target/data-matrix), the Gene Expression Omnibus repository (GSE125645, GSE97529, GSE59200 and GSE68379), ArrayExpress (E-MTAB-3610), and the PharmacoDB database (https://pharmacodb.pmgenomics.ca/). Source data for sample group memberships and average methylation values are included in Supplementary Data 1. All are other data are available from the corresponding author upon reasonable request.

## Code availability

All code used has been previously reported and is publicly available. The NCI BRB-ArrayTools v4.6.0, SPSS (version 24), CIBERSORTx^55,56^, R (version 3.5.1 and 4.1.0)^118^ with packages *ggplot2*^119^, *minfi*^40^, *amap*^120^*, bumphunter*^41^*, dendsort*^121^*, EnhancedVolcano*^122^*, ESTIMATE*^54^*, hmisc*^123^*, missMethyl*^57,58^*, pheatmap*^124^*, qqman*^125^*, RColorBrewer*^126^, and *survminer*^127^ were used for analysis.
