# Supplementary material for: Genome-wide DNA methylation patterns reveal clinically relevant predictive and prognostic subtypes in human osteosarcoma
Source: Commun Biol. 2022 Mar 8;5:213. doi: 10.1038/s42003-022-03117-1 (PMC8904843; doi:10.1038/s42003-022-03117-1)
Supplement: Supplementary file 1 — Supplemental Material [file 42003_2022_3117_MOESM1_ESM.pdf]

Genome-wide DNA methylation patterns reveal clinically relevant predictive and prognostic subtypes in human osteosarcoma.

Christopher E. Lietz[1], Erik T. Newman[1], Andrew D. Kelly[2], David H. Xiang[1], Ziying Zhang[1], Caroline A. Luscko[1], Santiago A. Lozano-Calderon[1], David H. Ebb[3], Kevin A. Raskin[1], Gregory M. Cote[4], Edwin Choy[4], G. Petur Nielsen[5], Benjamin Haibe-Kains[6], Martin J. Aryee[5], Dimitrios Spentzos[1*].

Supplementary Table 1. Genomic region enrichment in the methylation profiles. Enrichment (green) or depletion (red) significance as determined by the hypergeometric test. The Global profile was compared to all sites interrogated by the array, the RFS, CR, and Global profile differentially methylated sites were compared to the Global profile.

Region	Global (95% most variantly methylated sites) profile	Sites differentially methylated between the two main Global cluster groups (red vs blue)	Sites differentially methylated between the main Global cluster groups (orange vs red and blue)	RFS profile	CR profile
**CGI**	$1.91 x 10^{-53}$	$1.91 x 10^{-53}$	$5.94 x 10^{-22}$	$5.23 x 10^{-6}$	$1.39 x 10^{-4}$
**CGI $_{promoter\ associated}$**	$1.19 x 10^{-242}$	not tested	not tested	$8.97 x 10^{-3}$	$8.06 x 10^{-4}$
**Shore**	$1.03 x 10^{-3}$	$1.39 x 10^{-31}$	$4.26 x 10^{-11}$	0.0179	0.166
**Shelf**	$1.38 x 10^{-11}$	$5.67 x 10^{-28}$	0.378	0.522	0.020
**Open Sea**	$7.31 x 10^{-56}$	$5.09 x 10^{-200}$	$1.77 x 10^{-44}$	$4.48 x 10^{-9}$	$1.24 x 10^{-3}$
**Enhancer**	$1.33 x 10^{-51}$	$1.94 x 10^{-22}$	$2.49 x 10^{-51}$	$1.63 x 10^{-6}$	$5.83 x 10^{-3}$
**Intragenic**	$1.03 x 10^{-281}$	$1.52 x 10^{-27}$	$7.45 x 10^{-4}$	$2.59 x 10^{-3}$	$4.94 x 10^{-6}$

Supplementary Table 2. Associations (chi square test) between genomic alterations reported in the TARGET dataset and the methylation profile cluster groups.

Sample group	*TP53*		*RB1*		*CDKN2A*		*ATRX*	
	OR	p	OR	p	OR	p	OR	p
Global profile cluster 2 group	1.025	1.000	3.186	0.021	1.197	1	2.228	0.254
Global profile cluster 3 group	NA	0.888	NA	0.031	NA	0.400	NA	0.002
RFS profile cluster 2 groups	0.691	1	0.826	0.816	0.500	0.512	1.324	0.254
CR profile cluster 2 groups	1.025	1	0.569	0.255	0.791	0.757	0.596	0.405

Supplementary Table 3. Associations (t test) between methylation profile cluster groups or genomic alterations reported in the TARGET dataset and the CIMP scores.

profile / gene alteration	panel	p	p (promoter only)
Global	Toyota	5.2E-06	0.055
Global	Weisenberger	0.002	0.002
Global	Noushmehr	0.787	0.998
RFS	Toyota	0.420	0.584
RFS	Weisenberger	0.031	0.427
RFS	Noushmehr	0.993	0.238
CR	Toyota	1.5E-08	0.070
CR	Weisenberger	0.129	0.141
CR	Noushmehr	0.247	0.497
TP53	Toyota	0.898	0.444
TP53	Weisenberger	0.622	0.715
TP53	Noushmehr	0.960	0.788
MDM2	Toyota	0.781	0.544
MDM2	Weisenberger	0.676	0.854
MDM2	Noushmehr	0.898	0.946
RB1	Toyota	0.270	0.787
RB1	Weisenberger	0.701	0.415
RB1	Noushmehr	0.673	0.337
CDKN2A	Toyota	0.678	0.573
CDKN2A	Weisenberger	2.1E-04	3.5E-04
CDKN2A	Noushmehr	0.003	0.001
ATRX	Toyota	0.116	0.539
ATRX	Weisenberger	0.974	0.957
ATRX	Noushmehr	0.747	0.635

Supplementary Table 4. Differential methylation analysis of the methylation profiles in three independent clinical datasets. The fraction of all and differentially methylated (FDR < 0.1) CpG sites between the two primary cluster groups that were concordantly hypo / hyper methylated between the datasets are shown **(Fig. 5)**.

Profile	n sites	FDR < 0.1 in TARGET (%)	FDR < 0.1 in JNCCRI (%)	FDR < 0.1 in NY (%)	TARGET v. JNCCRI all site concordance (%)	TARGET v. JNCCRI significant site concordance (%)	TARGET v. NY all site concordance (%)	TARGET v. NY significant site concordance (%)	JNCCRI v. NY all site concordance (%)	JNCCRI v. NY significant site concordance (%)
Global	19264	56.9	6.1	0.0001	58.3	78.3	49.7	50.0	64.2	NA
RFS	374	97.9	7.8	13.6	79.1	100.0	86.4	98.0	77.1	100.0
CR	374	98.9	69.3	9.4	96.5	100.0	97.0	100.0	93.6	100.0

Supplementary Table 5. Associations (t test) between percent cellular tumor and CIBERSORTx predicted immune infiltration and the methylation profile and EPIMMUNE signature cluster groups.

cluster_groups	variable	mean_difference	p
G95 Fig.1b	%_cellular_tumor	-6.21	0.055
G95 Fig.1b	CIBERSORTx_predicted_absolute_immune_infiltration	0.05	0.648
RFS Fig. 3c	%_cellular_tumor	1.72	0.602
RFS Fig. 3c	CIBERSORTx_predicted_absolute_immune_infiltration	-0.04	0.708
CR Fig. 3d	%_cellular_tumor	4.33	0.184
CR Fig. 3d	CIBERSORTx_predicted_absolute_immune_infiltration	-0.06	0.534
EPIMMUNE Supplementary Fig. 6	%_cellular_tumor	-9.00	0.005
EPIMMUNE Supplementary Fig. 6	CIBERSORTx_predicted_absolute_immune_infiltration	0.21	0.035

Supplementary Table 6. Differential methylation comparison of the methylation profiles in the cell line GDSC and clinical TARGET datasets. The fraction of all and differentially methylated (FDR < 0.1) CpG sites between the two primary cluster groups that were concordantly hypo / hyper methylated between the datasets are shown (Fig. 9).

Profile	n sites	FDR < 0.1 in TARGET (%)	FDR < 0.1 in GDSC (%)	TARGET v. GDSC all site concordance (%)	TARGET v. GDSC significant site concordance (%)
Global	19264	56.9	16.0	76.8	99.7
RFS	374	97.9	0.8	80.2	100.0
CR	374	98.9	48.1	97.1	100.0

Supplementary Table 7. Spearman correlations between methylation profile sites and cell line aggressiveness metrics. The fraction of probes significantly ($p < 0.05$) correlated with the aggressiveness metrics and the fraction of 1,000 randomly generated lists with an equal or greater fraction of significantly correlated sites (permutation p) are presented.

		colony forming ability		invasion		migration		proliferation		tumorigenicity	
Profile	n	significantly correlated sites (p < 0.05) %	perm. p	significantly correlated sites (p < 0.05) %	perm. p	significantly correlated sites (p < 0.05) %	perm. p	significantly correlated sites (p < 0.05) %	perm. p	significantly correlated sites (p < 0.05) %	perm. p
RFS	374	4.8	0.920	5.9	0.189	6.4	0.054	12.3	< 0.001	2.9	1.000
CR	374	18.7	< 0.001	2.400	0.987	1.900	0.990	7.800	0.193	2.100	0.999

Supplementary Table 8. Spearman correlations between methylation profile sites and cell line response to standard chemotherapy. The fraction of probes significantly ($p < 0.05$) correlated with standard chemotherapeutics and the fraction of 1,000 randomly generated lists with an equal or greater fraction of significantly correlated sites (permutation p) are presented. MAP was not tested as a combination treatment, so combined MAP response was assessed by combining scaled methotrexate, doxorubicin, and cisplatin response metrics.

		cisplatin		doxorubicin		methotrexate		MAP score	
Profile	n	significantly correlated sites (p < 0.05) %	permutation p	significantly correlated sites (p < 0.05) %	permutation p	significantly correlated sites (p < 0.05) %	permutation p	significantly correlated sites (p < 0.05) %	permutation p
RFS	374	2.7	0.780	4.5	0.076	3.7	0.082	8.0	0.003
CR	374	1.1	0.999	3.5	0.401	2.700	0.435	3.200	0.912

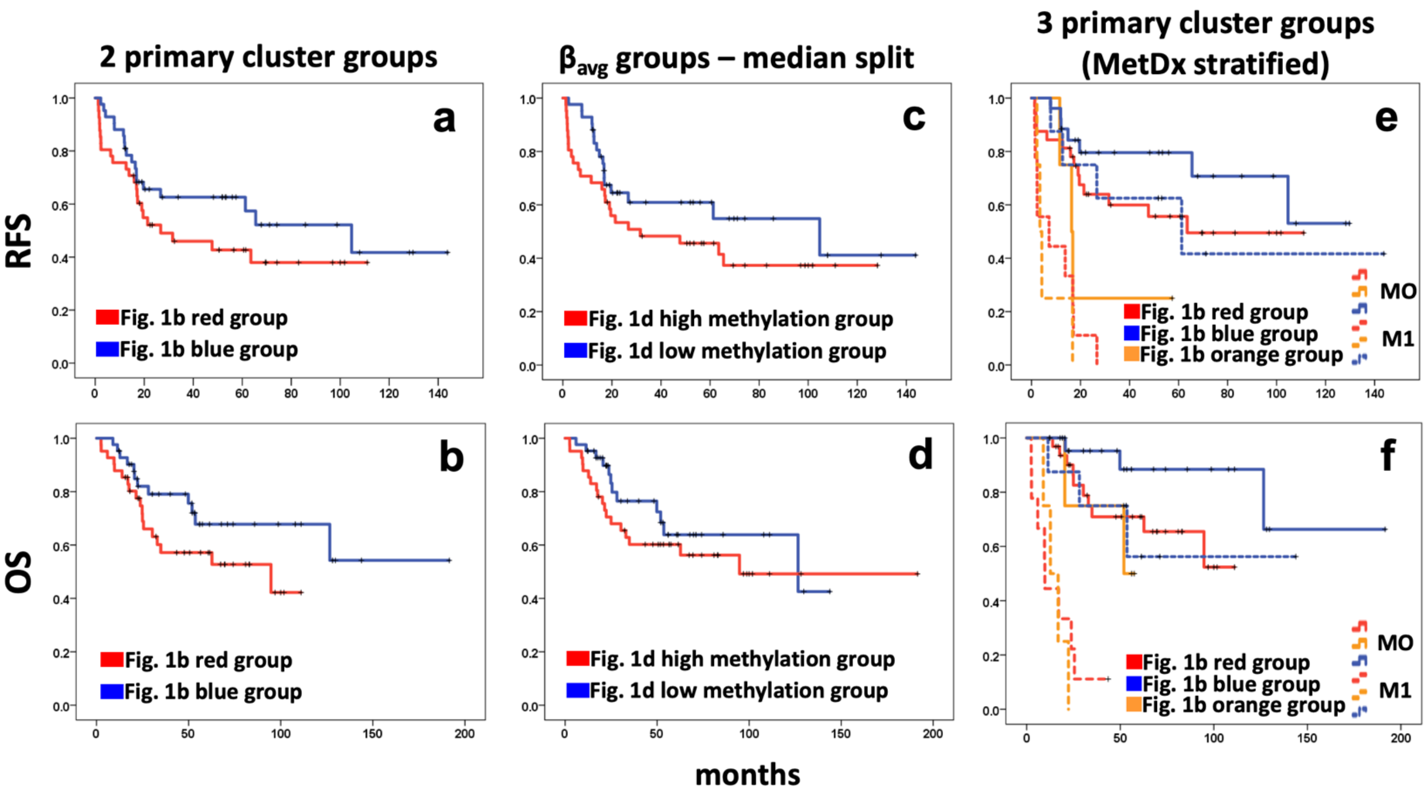

Supplementary Figure 1. Additional survival analysis of the Global profile hierarchical clustering and average methylation groups not shown in **Fig. 1**. Two main cluster groups: RFS (**a**, p = 0.176), OS (**b**, p = 0.106). Average β median split groups: RFS (**c**, p = 0.157), OS (**d**, p = 0.298). Three main cluster groups stratified for metastatic disease at the time of diagnosis: RFS (**e**, pooled p = 0.001), OS (**f**, pooled p = 0.001). Group colors correspond to those used in **Fig. 1**.

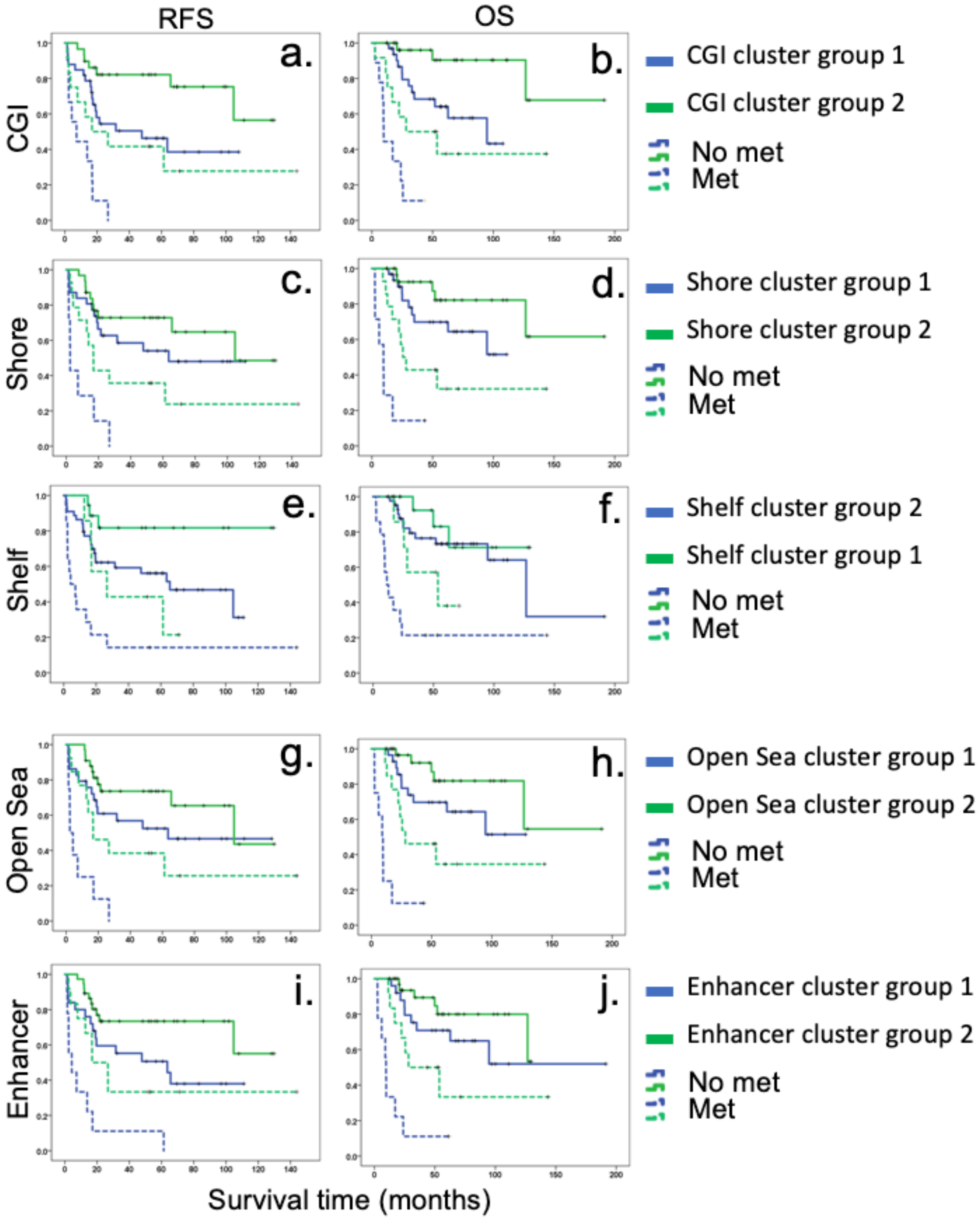

Supplementary Figure 2. Survival analysis of the two main hierarchical clustering groups generated using each of the genomic regions when stratified for metastasis at the time of diagnosis. CGI region: RFS (**a**, pooled p = 0.002) OS (**b**, pooled p = 0.001). Shore region: RFS (**c**, pooled p = 0.036), OS (**d**, pooled p = 0.005). Shelf region: RFS (**e**, pooled p = 0.006), OS (**f**, pooled p = 0.044). Open Sea region: RFS (**g**, pooled p = 0.011), OS (**h**, pooled p = 0.002). Enhancer region: RFS (**i**, pooled p = 0.003), OS (**j**, pooled p = 0.008).

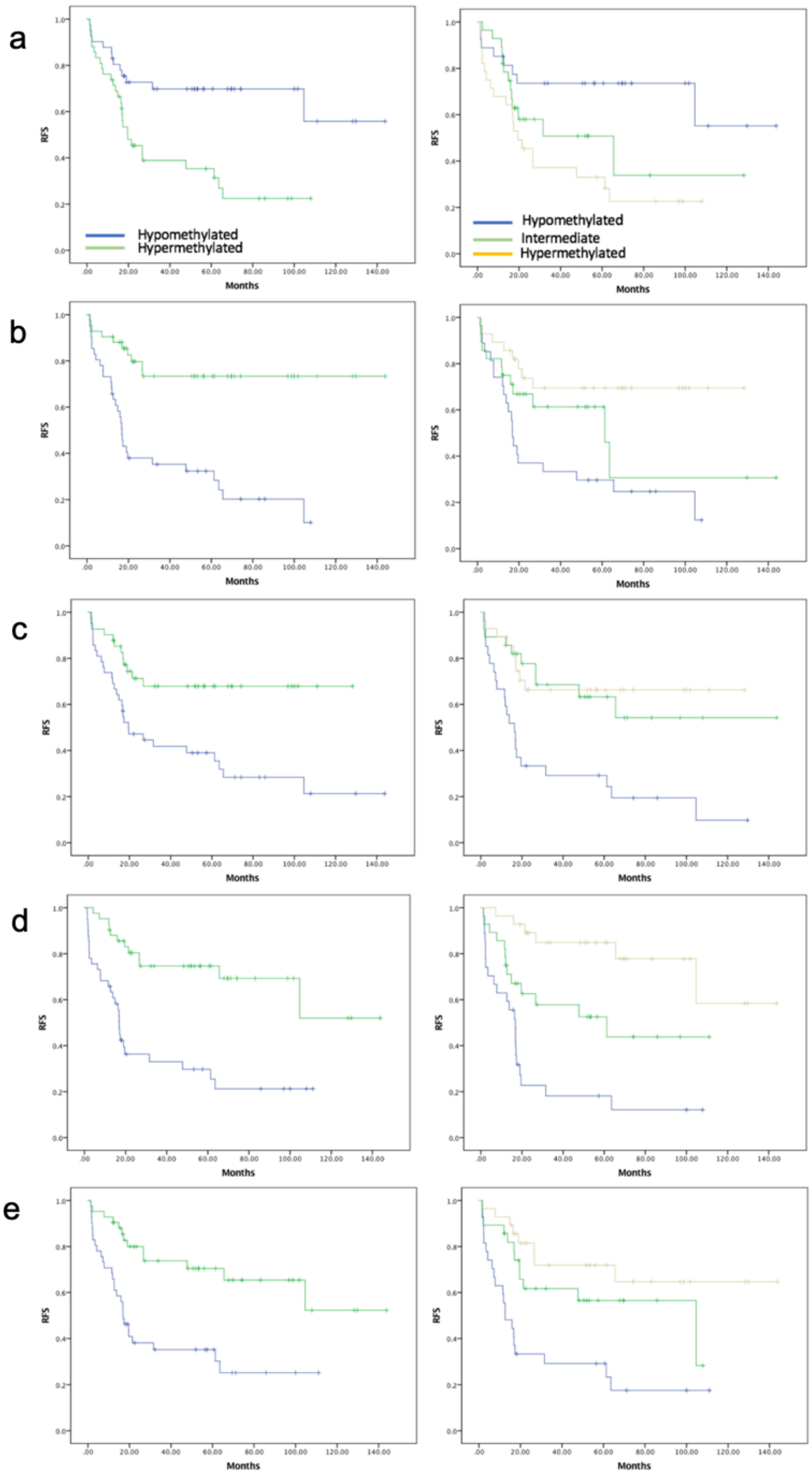

Supplementary Figure 3. RFS analysis of patient risk groups generated with methylation of single CpG site from each analyzed genomic region. Patient risk groups were generated by methylation level (median split and terciles). **a)** CGI region CpG site cg19848683 (*MECOM*), 2-group log-rank p value = 0.005, 3-group log-rank p value = 0.003. **b)** Shore region, CpG site cg04461028 (*HDAC4*), 2-group log-rank p value < 0.001, 3-group log-rank p value = 0.004. **c)** Shelf region, CpG site cg06835212 (*MEF2C*) 2-group log-rank p value = 0.002, 3-group log-rank p value < 0.001. **d)** Open Sea region, CpG site cg15595627 (*ANGPT1*), 2-group log-rank p value < 0.001, 3-group log-rank p value < 0.001. **e)** Enhancer region, CpG site cg12506775 (*TASOR2*), 2-group log-rank p value < 0.001, 3-group log-rank p value < 0.001.

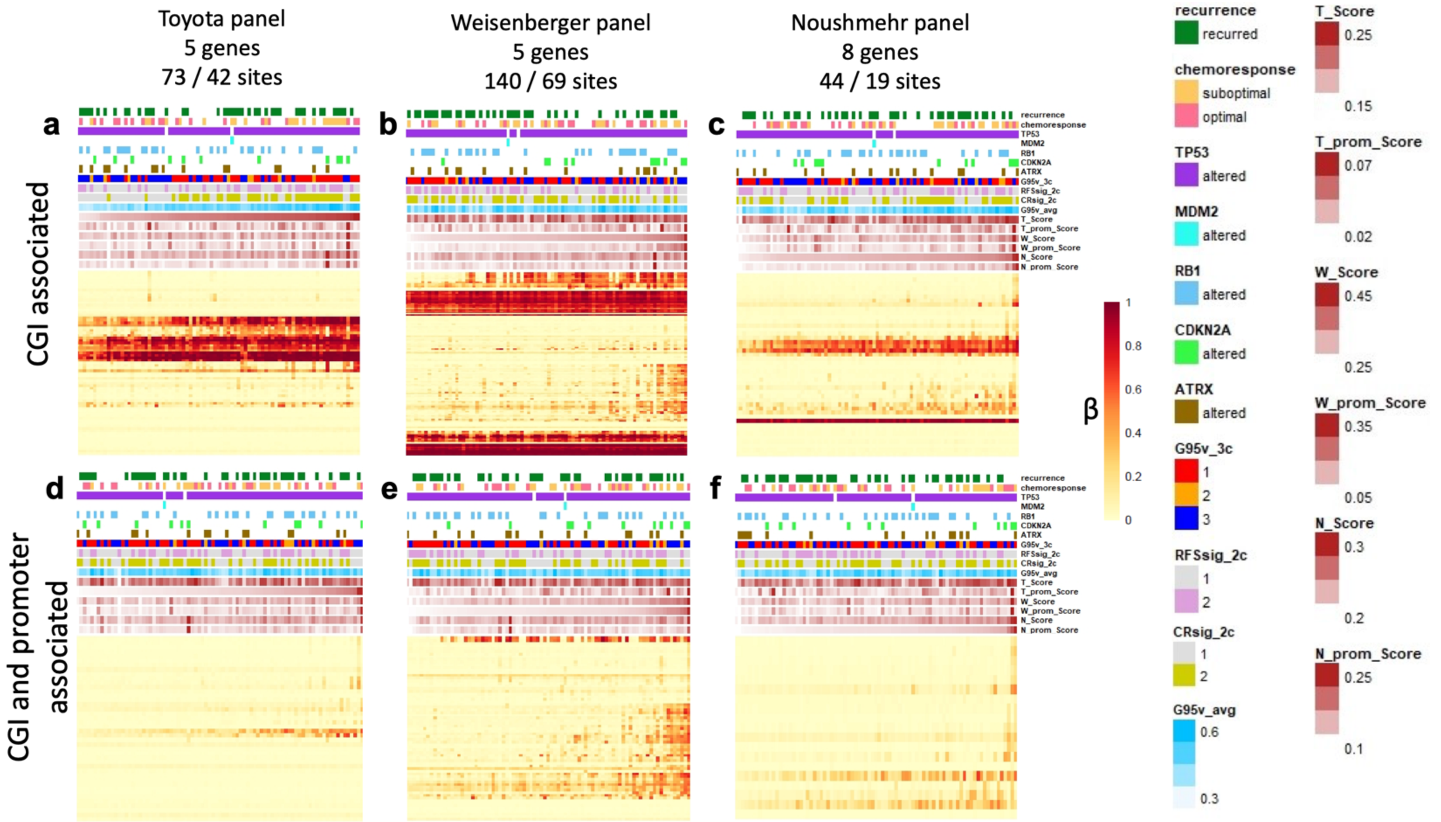

Supplementary Figure 4. Supervised CIMP panel heatmaps. Samples are ordered from lowest to highest CIMP score for each panel. **a**, **b**, and **c** use all CGI sites annotated to the Toyota, Weisenberger, and Noushmehr et al panels, respectively. **d**, **e**, and **f** use all promoter associated CGI sites annotated to the Toyota, Weisenberger, and Noushmehr et al panels, respectively.

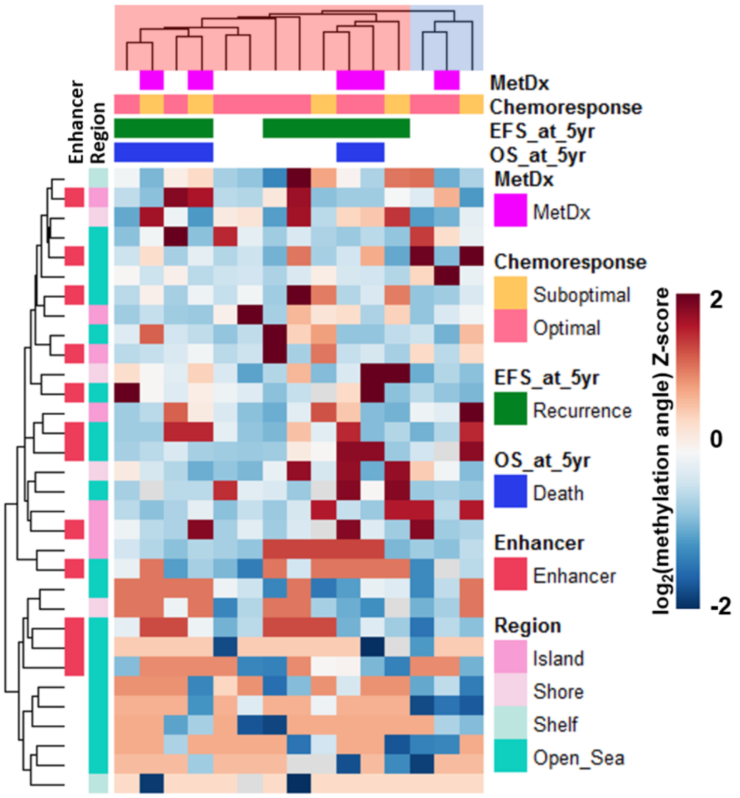

Supplementary Figure 5. Semi-supervised hierarchical clustering using the 32 of 374 RFS profile sites measured in the AECM dataset.

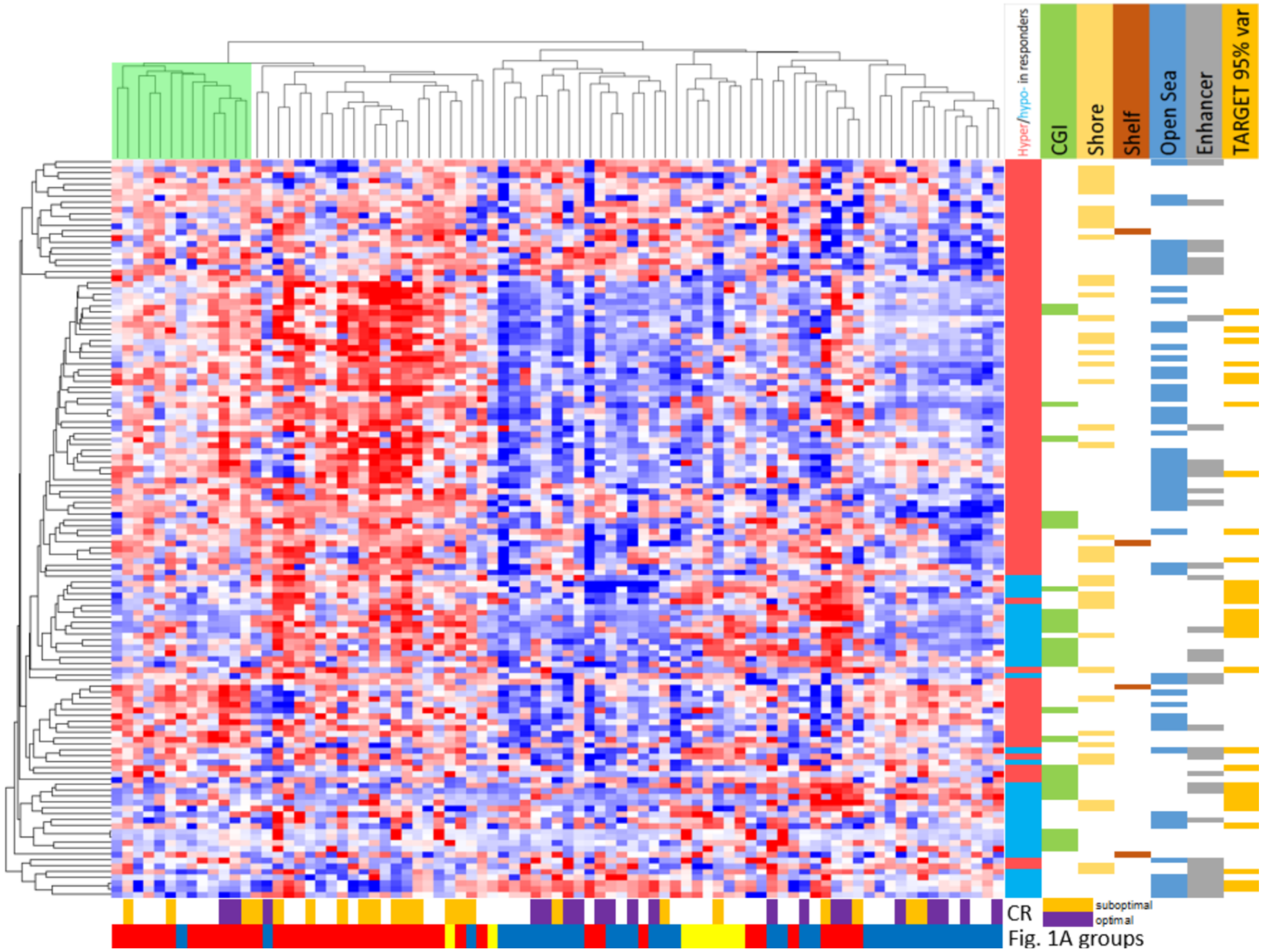

Supplementary Figure 6. Analysis of the EPIMMUNE lung cancer methylation signature. Hierarchical clustering and the heatmap were generated using M values of the 128 EPIMMUNE CpG sites mapped to the TARGET osteosarcoma dataset (two group cluster stability R index = 0.745). Green highlighted samples have a methylation profile predictive of optimal immune checkpoint inhibitor response in the EPIMMUNE discovery cohort. Sites hyper- (red) or hypo- (blue) methylated in immune checkpoint inhibitor responders in the EPIMMUNE study discovery cohort are displayed to the right of the heatmap (leftmost color bar). Genomic regions (middle color bars) and sites passing the 95% variance filtering criteria used for discovery analysis in the TARGET dataset (Global profile, rightmost color bar) are also displayed. Color bars below the heatmap annotate TARGET osteosarcoma sample response to chemotherapy, and cluster group membership from the Global clustering analysis **(Fig. 1b)**.

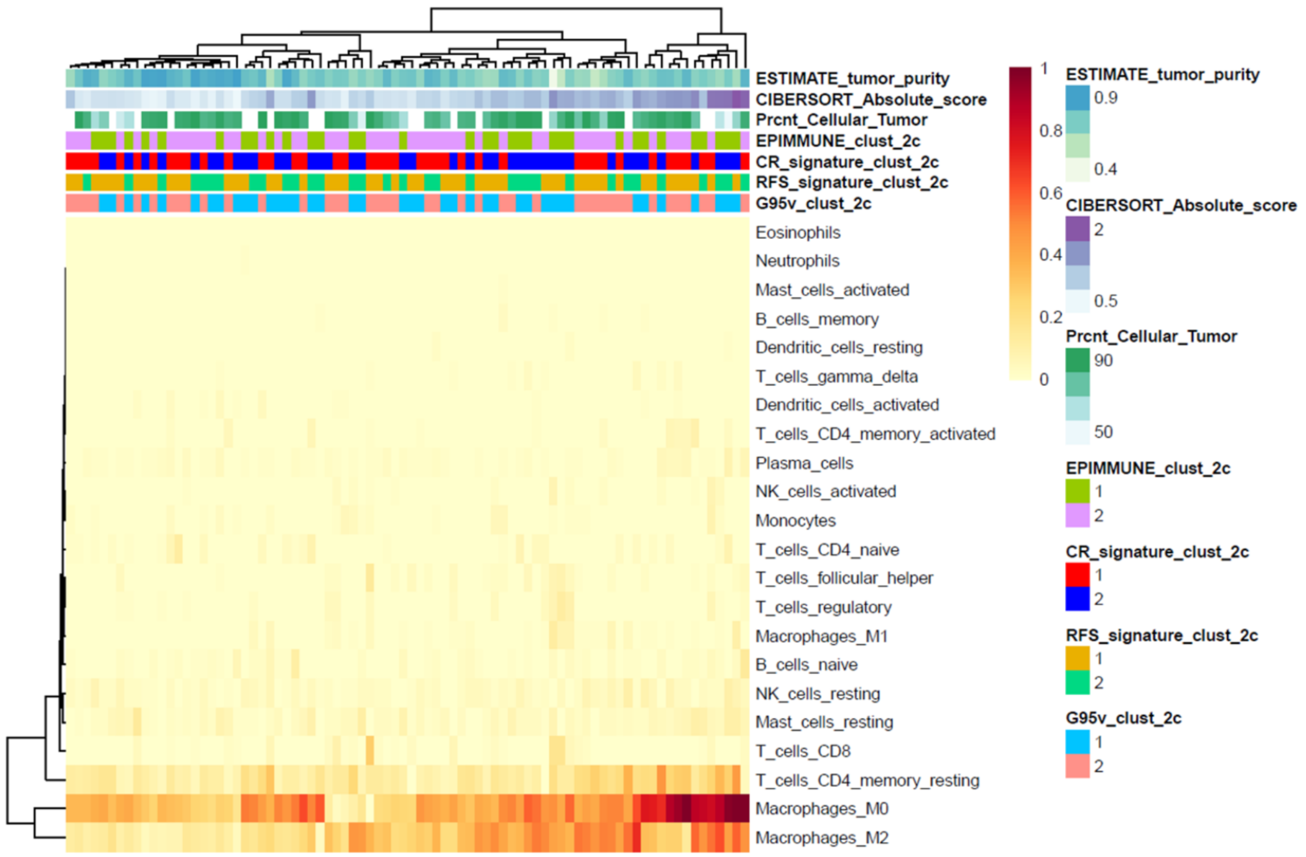

Supplementary Figure 7. Hierarchical clustering using the predicted infiltrate of 22 immune cell subtypes in the TARGET samples. Percent cellular tumor, ESTIMATE predicted cellular tumor, CIBERSORT absolute score, and sample methylation profile and EPIMMUNE signature cluster group memberships are annotated.

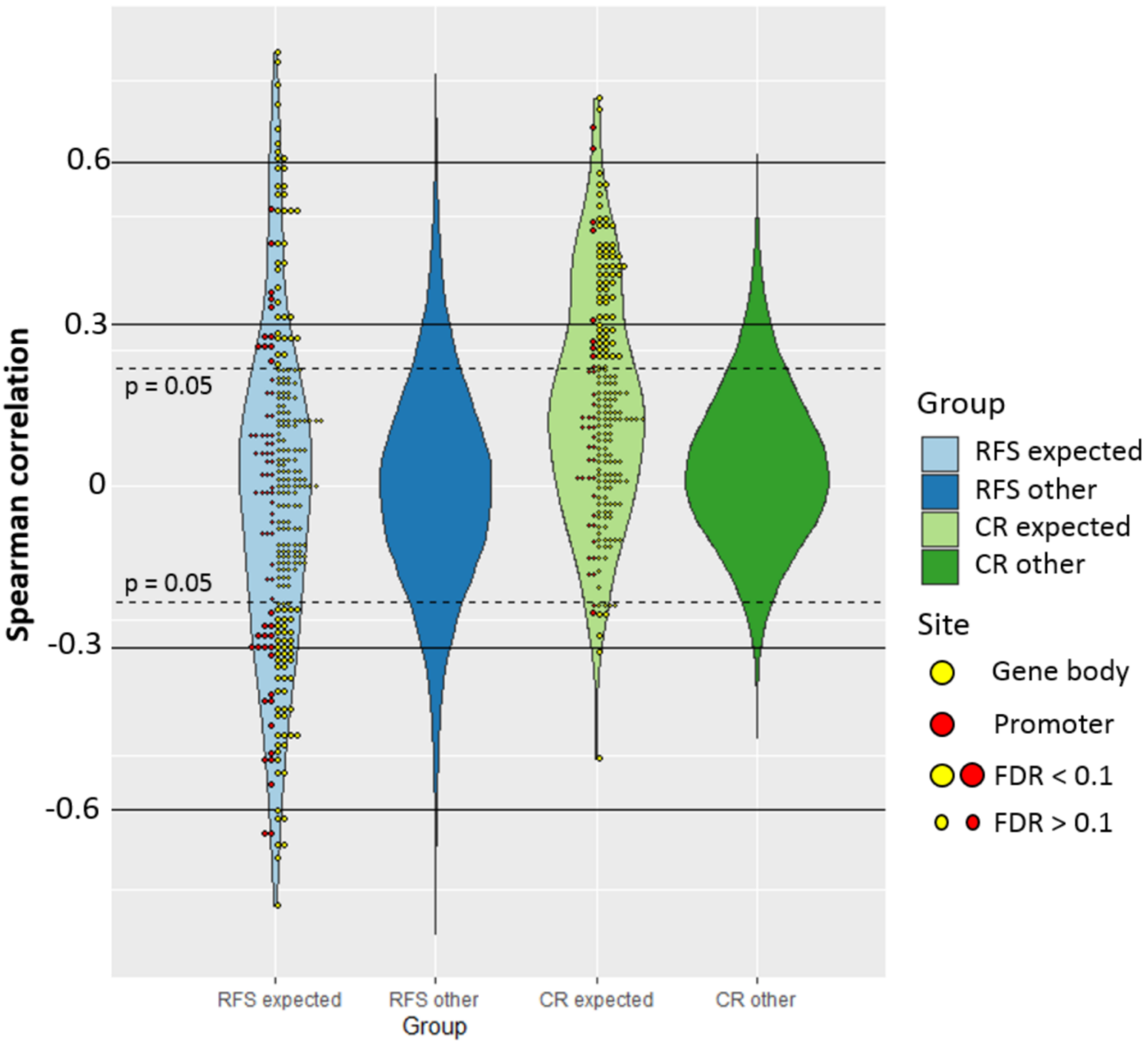

Supplementary Figure 8. Violin plots of the distribution of Spearman correlations between outcome profile methylation and gene expression. Correlations between annotated methylation – gene pairs (expected interaction) are compared to correlations between all other sites and genes in each profile. Promoter associated (red) and gene body (yellow) CpG sites are shown in different colors. Correlations between paired CpG sites methylation and gene expression with FDR < 0.1 are marked by enlarged points.

Supplementary Note 1.

Illumina Infinium HumanMethylation450 BeadChip data processing
To show that our results are not dependent on specific preprocessing methodology we processed the raw IDAT files for the TARGET dataset using the *minfi* package and functional normalization (*funnorm*) with default settings. CpG sites with detection p value > 0.01, those on the sex chromosomes, those with SNPs in the single base extensions or target CpG site, and those known to exhibit cross reaction with off target sites were removed[1]. We then repeated the main analyses with this alternatively processed dataset. We found a high degree of similarity with our prior results. Specifically, the list of 5% most variant sites in each version of the dataset was significantly overlapping (12,525 of 19,264 sites, $p < 1x10^{-300}$), the list of sites significantly associated with RFS (FDR < 0.1) was highly overlapping (834 of 885 sites, $p < 1x10^{-300}$), and the list of sites significantly associated with CR (FDR < 0.1) was highly overlapping (6,025 of 6,224 sites, $p < 1x10^{-300}$). The two patient cluster groups generated using the 5% most variant sites were very similar (Cramer's V = 0.930, $p = 2.4x10^{-17}$) between the two alternatively processed versions of the dataset.

Supplementary references

1. Chen, YA*, et al.* Discovery of cross-reactive probes and polymorphic CpGs in the Illumina Infinium HumanMethylation450 microarray. *Epigenetics* **8**, 203-209 (2013).
