# Supplementary material for: Genome-wide DNA methylation patterns reveal clinically relevant predictive and prognostic subtypes in human osteosarcoma
Source: Commun Biol. 2022 Mar 8;5:213. doi: 10.1038/s42003-022-03117-1 (PMC8904843; doi:10.1038/s42003-022-03117-1)
Supplement: Supplementary file 2 — Description of Additional Supplementary Files [file 42003_2022_3117_MOESM2_ESM.pdf]

Description of Additional Supplementary Files

File name: Supplementary Data 1

Description: Characteristics of the TARGET, AECM, JNCCRI, and NY clinical datasets and the GDSC in vitro dataset and sample level group memberships and risk scores from results presented in this report.

File name: Supplementary Data 2

Description: Methylation profile CpG sites and their statistical associations with clinical outcomes and sample groups.

File name: Supplementary Data 3

Description: Region based differential methylation analysis between the Global profile cluster groups using minfi bumphunter analysis. Regions were considered significantly differentially methylated if their area had a family wise error rate < 0.1 based on 1,000 permutations.

File name: Supplementary Data 4

Description: MissMethyl gometh GO and KEGG pathway enrichment in the methylation profiles.

File name: Supplementary Data 5

Description: Spearman correlation between the methylation profiles and transcription of genes annotated to the profile CpG sites and differential expression of the genes between the methylation profile cluster groups.

File name: Supplementary Data 6

Description: Geneset enrichment (GSA analysis) using the genes annotated to the methylation profiles. Pathways and terms with a LS permutation p value < 0.05 were considered significant.
